# Maternal gut microbiome interventions to improve maternal and perinatal health outcomes: Target product profile expert consensus and pipeline analysis

**DOI:** 10.1371/journal.pone.0321543

**Published:** 2025-07-02

**Authors:** Kate Mills, Joelle Tan, Tahlia Guneratne, Lindsay Keir, Maya Goldstein, Cécile Ventola, Maureen Makama, Anne Ammerdorffer, A. Metin Gülmezoglu, Joshua P. Vogel, Annie R. A. McDougall

**Affiliations:** 1 Maternal, Child and Adolescent Health Program, Burnet Institute, Melbourne, Australia; 2 Impact Global Health, London, United Kingdom; 3 School of Public Health and Preventive Medicine, Monash University, Melbourne, Australia; 4 Concept Foundation, Geneva, SwitzerlandBangkok, Thailand; 5 Monash Institute of Pharmaceutical Sciences, Monash University, Parkville, Australia; Beni-Suef University, EGYPT

## Abstract

**Objective:**

To develop a novel Target Product Profile (TPP) outlining the minimum and optimal requirements of probiotics targeting the maternal gut microbiome, create a research and development (R&D) pipeline of maternal microbiome interventions, and identify the highest potential probiotic candidates matching TPP criteria.

**Design:**

A mixed-methods study including in-depth interviews, an international survey, and online public consultation, with systematic R&D pipeline development.

**Setting:**

International research context in maternal gut microbiome interventions.

**Population:**

Ten stakeholder groups were included in the study for feedback on the TPP development.

**Methods:**

Stakeholder feedback from 23 interviews and 32 survey responses was analyzed to revise the TPP. A systematic search of databases (Adis Insight, ClinicalTrials.gov, WHO ICTRP, Ovid MEDLINE, and relevant grant databases) identified drugs, supplements, and biologics targeting the maternal gut microbiome. Probiotic candidates were matched against key TPP criteria to identify promising options for future research.

**Main Outcome Measures:**

Stakeholder consensus (≥75% agreement) on TPP variables and identification of high-potential probiotic candidates.

**Results:**

The TPP met consensus for most of the 20 variables: 16 for minimum and 14 for optimal targets. Interviews raised issues concerning indication, target population, diagnostic requirements, and efficacy outcomes. Of 38 candidates identified in the maternal microbiome pipeline (2000–2023), eight were probiotics, with one high-potential candidate (*Vivomixx*) and two medium-potential candidates (*Lactobacillus spp. and Bifidobacterium spp.*) identified.

**Conclusions:**

This study produced the first TPP and pipeline analysis for maternal gut microbiome interventions, identifying probiotics with higher potential. Few candidates reached late-phase research, highlighting the need for efficacy trials.

## Introduction

Maternal gut microbiome conditions have been associated with adverse pregnancy outcomes [1,2]. While this relationship is complex and not yet entirely understood, with limited prevalence data available, it is hypothesised that pregnant women in settings with poor sanitation, high pathogen exposure, and chronic infections may be more likely to have environmental enteric dysfunction (EED) [3–12]. EED is characterised by gastrointestinal dysbiosis and chronic low-grade inflammation in the small intestine, caused by subclinical enteropathogen infection [13]. This leads to altered gut morphology and impaired barrier function, resulting in poor macro/micronutrient absorption capacity [13]. Maternal EED appears to increase the likelihood of stillbirth, miscarriage, foetal growth restriction, preterm birth, small-for-gestational-age babies, and poor neonatal outcomes [14].

The use of microbial interventions targeting the maternal gut microbiome to address EED is an active research area; currently, no specific treatments are clinically recommended for EED in pregnant women or the general population [2,15]. Much of the current research on EED focusses on children, particularly in relation to stunting and diarrhea [1,16,17]. Probiotics, defined as “live microorganisms which when administered in adequate amounts confer a health benefit on the host”, can be used as a potential therapy to target the maternal gut microbiome [18]. Probiotics may be regulated in two ways – as supplements for general health benefits or as drugs to treat a specific condition or disease [19]. If probiotics were able to resolve or mitigate EED in pregnancy, this could theoretically optimise maternal nutritional absorption and lead to improved maternal and newborn health outcomes.

The Accelerating Innovation for Mothers (AIM) project, established in 2020, seeks to encourage investment in research and development (R&D) to address the lack of efficacious and accessible medicines and technologies for pregnancy-related conditions. To achieve this we adopted a multi-faceted approach, including development of the first target product profiles (TPPs) for maternal medicines, diagnostics and devices [20,21], and creating comprehensive pipelines of maternal medicines, diagnostics and devices R&D pipelines [22]. TPPs guide R&D stakeholders – such as donors, researchers, developers, manufacturers, innovators and regulators – to better accelerate their R&D efforts by defining the clinical and public health requirements for novel medical products [23,24]. We have also assessed candidates in these R&D pipelines against criteria in TPPs to identify high-potential medicines, diagnostics and devices on which to focus future R&D efforts to meet the needs of pregnant women with these conditions [25–27].

Currently, no TPPs or comprehensive R&D pipelines exist for maternal gut microbiome interventions. This study aimed to 1) develop the first TPP for maternal gut microbiome-altering drugs and supplements, specifically probiotics, to elicit improvements in maternal outcomes, 2) create an R&D pipeline of these interventions in clinical or preclinical development between 2000–2023, and 3) compare candidates in the clinical R&D pipeline to the TPP to identify the most promising candidates for further research.

## Methods

### Target product profile development

**Fig 1:**
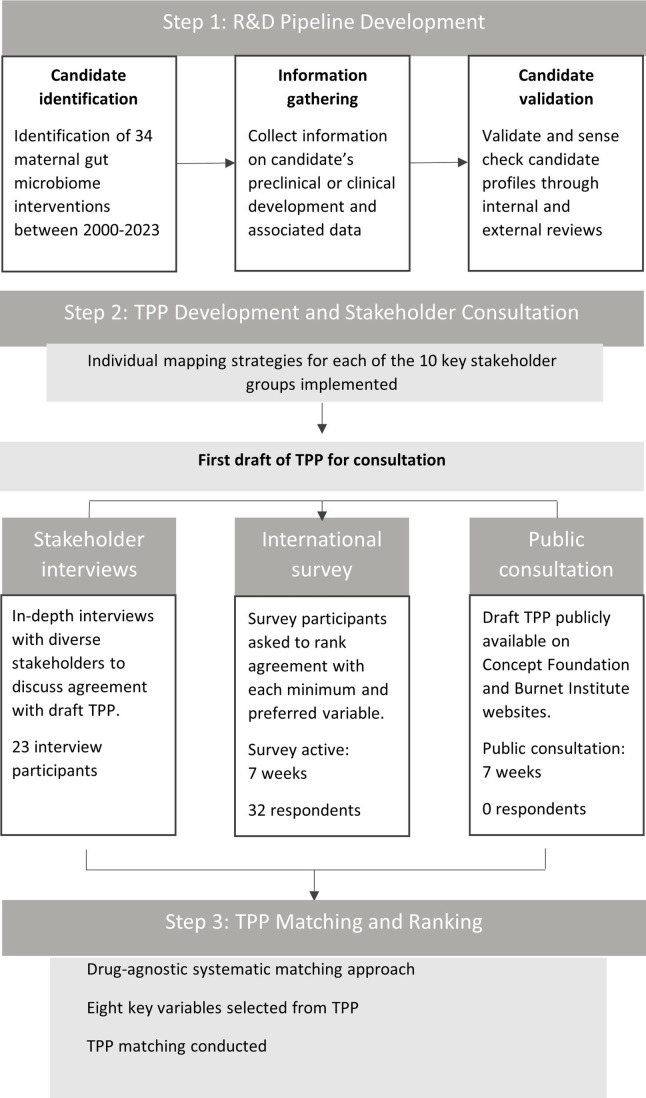
Schematic of TPP development process.

We prepared a study protocol for TPP development guided by the process outlined by Lewin et al [28], and informed by our previous TPPs for medicines for preeclampsia [20] and preterm birth [21] (Fig 1). The study protocol was reviewed and approved by the Alfred Ethics Committee for Human Research (project number 32/23).

Previous TPP templates from the AIM project, the World Health Organization (WHO) and other international organisations were reviewed to determine what criteria to include [20,21,29,30]. An initial TPP draft was developed based on extensive literature reviews and discussions with maternal health and microbial interventions subject matter experts. Subsequently, we conducted a comprehensive stakeholder mapping process to ensure diverse participation from ten key stakeholder groups: obstetricians, midwives/nurses, academic researchers, dieticians/nutritionists, antenatal care/nutrition programme managers, biotech and nutraceutical manufacturers, consumer representatives, guideline panel members, procurement experts, and international health agency/organisation staff. Systematic identification and snowball sampling were used to identify stakeholders (S2 Table).

#### International stakeholder survey.

An international online stakeholder survey was conducted between 20^th^ August and 9^th^ October 2023 using the survey platform Qualtrics [31]. Respondents rated their agreement with the intended use case scenario and characteristics for each variable (minimum and optimistic) using a 5-point Likert scale. Free-text comments could also be added. The survey and invitation email were available in English, French and Spanish.

Survey invitations were sent to maternal and perinatal health professionals and stakeholders from ten key groups (S2 Table). Efforts were made to ensure respondent diversity in location, gender, and professional role. The survey was also distributed through clinician-researcher networks and email distribution lists. The survey link was anonymous, and recipients were encouraged to forward it to other relevant stakeholders, so an exact response rate cannot be calculated. Consensus for any variable was pre-defined as at least 75% of respondents either agreeing or strongly agreeing.

#### In-depth stakeholder interviews and public consultation.

In parallel with the stakeholder survey, we conducted in-depth, one-on-one interviews with key stakeholders. Diverse perspectives were ensured by sampling participants from each of the ten stakeholder groups, as well as a range of respondents from different geographical location, country income level, and gender (S2 and S3 Tables). These interviews sought feedback on the draft TPP, focusing on background information, use-case scenario, and variable characteristics (minimum and optimal). A semi-structured guide was used, with interviews emphasising variables relevant to the expertise and perspective of each interviewee.

Between 24^th^ July and 11^th^ September 2023, we invited 109 stakeholders to participate, with 23 agreeing to take part. Stakeholders were contacted via email and provided with a copy of the TPP to review prior to the interview. Conducted via Zoom or Teams by an AIM project researcher (KM), each interview lasted up to 60 minutes and was conducted in English. Informed consent was obtained at the beginning of each interview, including consent to record the interviews to supplement notes captured during the interview. The draft TPP was also made available online between 20^th^ August and 9^th^ October 2023 via the Burnet Institute and Concept Foundation websites for public comment. Feedback was requested to be shared via email with the research team.

#### Synthesis, analysis and finalisation.

Stakeholder interview outputs were analysed using qualitative content analysis to identify major and minor issues, and key themes were summarised [32]. Additionally, barriers to effective product development or implementation were identified. Leveraging our previous experience in TPP development, directed content analysis was used to establish an initial coding framework and summative content analysis for counting and comparison of major and minor issues. Excel spreadsheets were used to collate and categorise all major and minor feedback for analysis.

Survey responses were analysed to determine whether consensus was reached for each variable. Written comments, especially on variables that did not reach consensus, were reviewed. Variables that did not meet consensus or with strong disagreement from interviews were discussed and modified in the TPP by the research team. All findings informed revisions to the draft TPP, including changes to the use case scenario (Box 1). The final draft was reviewed by the AIM project team before finalisation and publication (S1 Table).

**Box 1. Final use case scenario in the TPP for probiotic interventions targeting the maternal gut microbiome**

A probiotic supplement or drug that targets the maternal gut microbiome in pre-conception, pregnancy and/or lactation. The product should impact at least one of the following: maternal gut and systemic inflammation, gut permeability, pathogen burden or microbiome composition or functions that are linked to maternal (e.g., gestational diabetes, hypertension, obesity, preeclampsia, maternal infection) and/or infant (e.g., small for gestational age, preterm birth, low birth weight, sepsis, necrotising enterocolitis, wasting, stunting) outcomes. Interventions should be affordable, and self-administered non-invasively.

### R&D pipeline development

The pipeline mapping strategy was designed to capture all drugs, dietary supplements and biologic candidates targeting the maternal gut microbiome in preclinical and clinical development. This approach, detailed in previous publications [25,27,33,34], involved a systematic search of relevant databases to identify all relevant candidates – this included Adis Insight, ClinicalTrials.gov, WHO International Clinical Trials Registry Platform (ICTRP), Ovid MEDLINE and several grant databases. To be included, candidates had to meet the following inclusion criteria: a) intended to prevent or treat maternal EED; b) specifically tested in, indicated for or targeted for use in pregnant women and/or within the postpartum period; c) indicated for improvement of maternal outcomes or neonatal outcomes; d) either in active discovery/preclinical or clinical development now, or have been in development at one point between 2000 and 2023, or approved and registered for clinical use and/or used currently in clinical treatment (off-label); and e) either entirely new entities, existing/repurposed/label extensions, or new formulations or dosing of existing/registered products. Eligible candidates were compiled into a pipeline database summarising key information on each candidate, including product type, stage of R&D development, and an overview of trials conducted (S4 Table; https://www.policycuresresearch.org/maternal-health-pipeline/). Candidate profiles could also be filtered by a range of characteristics including active/inactive status, repurposed/new, and clinical use status.

### Identifying high-potential candidates within the R&D pipeline

To evaluate the potential of R&D pipeline candidates based on the TPP, we applied an agnostic, systematic matching approach [25,26]. We used eight key variables from the TPP as ranking criteria for all probiotic candidates in clinical development (S5 Table). These variables were deemed most important for real-world implementation, informed by our previous TPP matching studies [25,26]. TPP matching was conducted by two independent reviewers (KM, TG), with any differences resolved through discussion with a third reviewer (AM). Preclinical candidates were not matched due to limited available information at this early stage of development.

Candidates were assigned a numerical score indicating the degree of alignment with each TPP variable. Variables such as efficacy and safety were accorded higher weighting (S5 Table). Matching scores were visually presented with candidates classified as meeting optimal criteria (dark green), meeting minimum criteria (light green), partially meeting minimum criteria (yellow), not meeting minimum criteria (red), or not yet known (grey). This systematic evaluation allowed classification of candidates as high, medium, or low potential based on available evidence.

## Results

### Target product profile development

#### Survey and public consultation.

Thirty-two survey responses were received, across all WHO geographical regions (S3 Table) – Western Pacific (15; 46.9%), Europe (6; 18.8%), the Americas (5; 15.6%), Africa (3; 9.4%), South-East Asia (2; 6.3%), and Eastern Mediterranean (1; 3.1%). There was gender diversity among the survey respondents – 20 women (63%), 11 men (34%), and 1 preferring to self-describe their gender (3%). Respondents included academic researchers (18; 50.0%), nurses/midwives (5; 13.9%), obstetricians (4; 11.1%), international health agency or organisation staff (4; 11.1%), international guideline panel member (1; 2.8%), staff of microbiome or nutritional product companies (1; 2.8%), maternity health services manager (1; 2.8%), and other (2; 5.6%). One respondent disclosed a potential commercial or financial conflict of interest.

**Fig 2:**
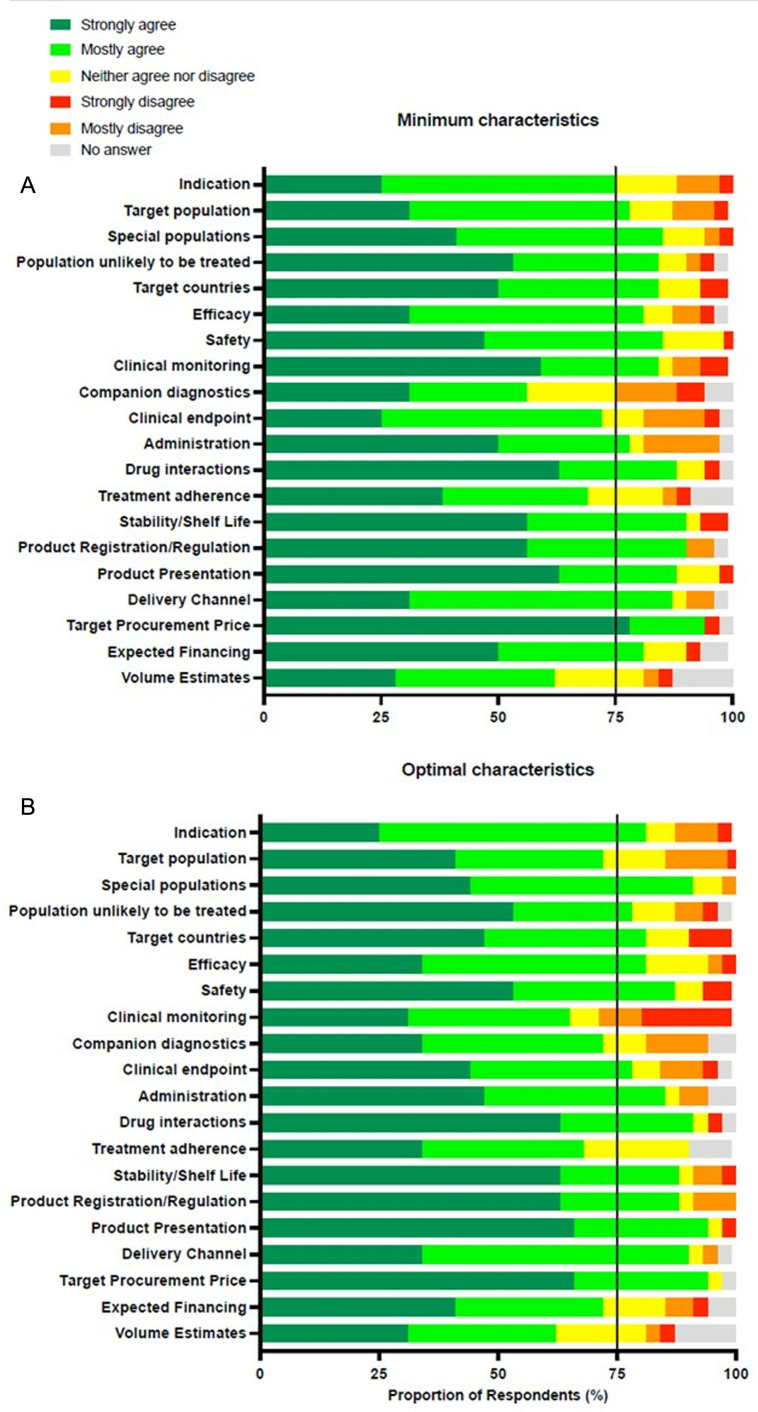
*Survey* *responses*. Results from international stakeholder survey. Percentage of respondents that strongly agreed (dark green), mostly agreed (light green), were neutral (yellow), mostly disagreed (orange), strongly disagreed (red) or did not respond (grey) in response to the minimum and preferred variables in the TPP. Consensus was considered agreement equal to or greater than 75% (black line).

The online survey met the 75% or more consensus threshold for the majority of the 20 TPP variables – 16 for minimum and 14 for optimal targets (Fig 2). Agreement was less than 75% for both minimum and optimal targets for three variables – companion diagnostics, treatment adherence and volume estimates. The clinical endpoint variable did not meet consensus for the minimum target only. Consensus was not met for the optimal target of target population, clinical monitoring, and expected financing sources. Written comments explained rationales for disagreement on these variables. No responses were received via the online public consultation webpages.

#### Stakeholder interviews.

In-depth interviews were conducted with 23 expert stakeholders (16 females and 7 males), representing various WHO regions (five from Africa, five from the Americas, five from South-East Asia, three from Europe, three from Western Pacific and two from Eastern Mediterranean) – 57% from low- or middle-income countries and 43% from high-income countries (S3 Table). Interview participants were included from all ten key stakeholder groups.

Findings from both the survey and interviews were broadly aligned. Major themes included discussions on indication and target population, with consensus that interventions should focus on population rather than individual level due to a current lack of EED diagnostics. This was also reflected in several survey comments. Key population-level risk factors such as poor sanitation, high environmental pathogen burden and recurrent infections were highlighted for targeting populations for probiotic supplements. However, unlike probiotic supplements, probiotic drugs would remain specifically indicated for EED at the individual level. Several interviewees noted the importance of including overweight or obese women, as well as those who are undernourished.

Most interviewees expressed disagreement with initial iterations of the efficacy outcomes which focused solely on measures of gut inflammation, noting the complexities of EED pathogenesis and lack of consensus on a validated diagnostic test. Many stakeholders were uncertain regarding what constituted a clinically significant difference in EED, given the incomplete state of current knowledge on how a healthy gut microbiome should be defined, as well as variations in gut microbiome composition across populations.

Minor themes included consideration of prebiotics or synbiotics (a combination of prebiotics and probiotics) in the background information, and basing volume estimations on the prevalence of risk factors for EED (such as poor water, sanitation and hygiene, and undernutrition), rather than EED incidence, which is currently not known. Two interviewees raised concerns about the safety of probiotic use in pregnant women, based on a 2021 Cochrane review [35] that reported a potentially increased risk of pre-eclampsia in overweight and obese women taking probiotics.

#### Finalisation of Target Product Profile.

The author group refined the indication to distinguish between probiotic supplements for gut health and probiotic drugs for treating EED. The target population was amended to a population-level focus in settings with increased risk of EED. Clinical efficacy outcomes were expanded to include systematic inflammatory markers, reduced enteropathogen abundance, improved pregnancy or birth outcomes, improved breastmilk composition, and improved infant growth or health outcomes. Probiotic supplements no longer required diagnostic testing, whereas this remained a requirement for probiotic drugs. A label claim variable was added in place of clinical endpoint for licensure, as this was deemed more relevant, and the treatment adherence variable was removed. Volume estimates were updated to reflect the population-level focus of the TPP, replacing individual EED diagnosis. Full details of the final TPP can be found in S1 Table.

### Mapping and analysis of the R&D maternal microbiome medicines pipeline

**Fig 3:**
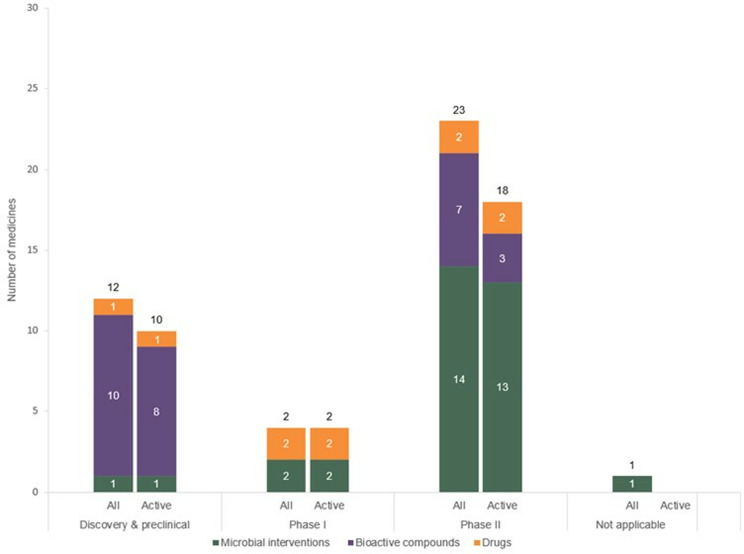
Maternal enteric microbiome medicines by R&D stage, product type and development status (active vs inactive).

Between 2000 and 2023, 38 candidates were identified as part of the complete maternal microbiome interventions pipeline (S6 Table). None of the 38 candidates were approved for use for maternal gut microbiome-related indications. Most (30; 79%) were in active development, with evidence of R&D activity within the last three years, while the remaining eight candidates (21%) were inactive (Fig 3). Of the 38 candidates, twelve (32%) were in the discovery or preclinical phase, two (5%) in Phase I, 23 (60%) in Phase II, none (0%) in Phase III, and one (3%) (a fermented soy and dairy product) was not specified. Eleven candidates (29%), most of which were traditional fermented foods, were ‘region specific’ – that is, originating from a specific country or geographical region. Nearly all candidates (32; 84%) were repurposed rather than new biological or chemical entities.

**Fig 4:**
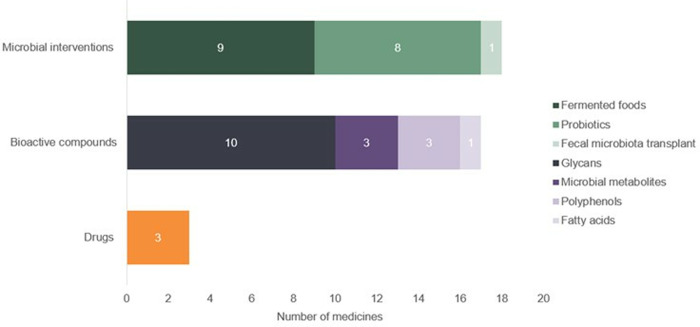
Maternal enteric microbiome medicines by product type and subtype (active and inactive).

Nearly half (18; 47%) were microbial interventions, followed by bioactive compounds (17 candidates (45%); glycans, polyphenols, fatty acids or microbial metabolites), and small-molecule drugs (3 candidates (8%)) (Fig 4). Of the 18 microbial interventions, nine were fermented foods, eight were probiotic supplements and one was a faecal microbiota transplant. Details of the eight probiotic candidates can be found in Table 1.

**Table 1:** Details of probiotic candidates from R&D pipeline. Full details of all candidates in the pipeline, including related literature, is available at https://www.impactglobalhealth.org/data/maternal-health-pipeline.

Name	Product type	Product subtype	Priority ranking
*Vivomixx*	Microbial interventions	Probiotics	High
*Lactobacillus spp.*	Microbial interventions	Probiotics	Medium
*Bifidobacterium spp.*	Microbial interventions	Probiotics	Medium
Probiotic combination – four unspecified strains	Microbial interventions	Probiotics	Low
Probiotic combination – unspecified strains	Microbial interventions	Probiotics	Low
Probiotics and LC-PUFA – combined, unspecified strains	Microbial interventions	Probiotics	Low
*Lactobacillus* and *Bifidobacterium* – combined	Microbial interventions	Probiotics	Low
*Akkermansia muciniphila*	Microbial interventions	Probiotics	N/A - preclinical

### Target product profile matching of candidates

Seven of the eight probiotic supplements were in clinical development and thus were matched against key TPP variables. These candidates included one in Phase I and six in Phase II. None had efficacy evidence, as no trials in pregnant women with or at risk of EED had been completed; one trial involving women with EED was ongoing [36]. Among these candidates, one was assessed as high potential, two as medium potential and four as low potential (S5 Table).

The high potential candidate was *Vivomixx,* consisting of eight probiotic strains. This candidate met either minimum or optimal characteristics for six variables – target population, target country, companion diagnostic, clinical monitoring, safety and format/administration. A Phase II trial is ongoing in Senegal with 76 pregnant women with EED [36]. Additionally, a completed study in Denmark including 49 obese pregnant women (not tested for EED) investigated the effects of this formulation on gestational weight gain and maternal glucose homeostasis [37]. However, the study concluded larger trials are needed to evaluate pregnancy-related outcomes. There is currently insufficient information to assess the remaining key variables (efficacy and stability).

Two candidates were ranked as medium potential. *Lactobacillus spp.* met optimal characteristics for three variables – target country, format/administration, and stability – and partially met the minimum criteria for target population. This was due to evidence from two clinical trials in pregnant women, with one completed (but no published results available) in Sweden [38] and one ongoing in Ghana [39]. *Bifidobacterium spp.* met optimal characteristics for four variables, including target country, safety, formulation/administration, and stability. This was based on evidence available from two clinical trials exploring *Bifidobacterium spp.,* both of which investigated strain transfer from pregnant women to infants – one completed in Ireland [40] and one ongoing in China [41].

Four probiotics combinations *(Lactobacillus and Bifidobacterium – combined, Probiotic combination – four unspecified strains, Probiotic combination – unspecified strains, and Probiotics and LC-PUFA – combined, unspecified strains)* were ranked as low potential candidates. *Lactobacillus and Bifidobacterium – combined* met optimal characteristics for two variables – format/administration and stability – and partially met for target country. Data exists from two clinical trials for this candidate, with a study in the US not yet publishing results, and a study in China being halted due to recruitment difficulties [42,43]. All other variables had insufficient information available to assess. The remaining three low potential candidates had only one clinical trial registered each [44–46], and no published results were available.

## Discussion

### Main findings

We have developed the first R&D pipeline and publicly available TPP for interventions targeting the maternal gut microbiome. Using a mixed-methods approach with inputs from a diverse international sample of stakeholders, our study found agreement among participants for most TPP variables. Participants largely agreed that 1) to be effective, probiotic interventions should be implemented at a population level unless an appropriate diagnostic test for EED is developed; 2) there should be a special focus on low-resource settings where the burden of EED is likely greatest; 3) the safety of any product must be guaranteed before implementation and scale-up; 4) product stability without any need for cold chain is required; and 5) products must be affordable, particularly in low- and middle-income countries (LMICs). Additionally, stakeholders believed that a broader set of clinical efficacy outcomes is required in future research, given the diversity of health effects that may be associated with targeting the maternal gut microbiome [47]. The R&D pipeline for candidate medicines targeting the maternal gut microbiome found few candidates overall. However, despite the early stage of R&D for this condition, of the candidates identified a high proportion were active, reflecting increasing interest in this field in recent years. Eight out of 38 candidates were probiotics, and only one of these is currently being investigated in a clinical trial specifically for EED in pregnant women.

### Interpretation, in light of known evidence

Stakeholder feedback was dominated by whether the intervention should target women with EED specifically or be applied population-wide. This reflects the current challenge in diagnosing EED due to the absence of an agreed, validated diagnostic test for pregnant women [48]. While an intestinal biopsy via endoscopy is the gold standard for diagnosing EED, it is not recommended during pregnancy due to potential safety concerns [49,50]. Ideally, a non-invasive diagnostic test would be developed to test pregnant women for EED, however achieving this will require innovative R&D advancements. Many nutritional deficiencies during pregnancy are addressed with population-level strategies, in part due to diagnostic challenges. For example, calcium supplementation for pregnant women in settings with low dietary calcium intake to prevent hypertensive disorders, and folic acid intake to reduce risk of neural tube defects [51], are both population-level health strategies, as opposed to testing and treating individual women for deficiencies. This approach removes the need for individual diagnostics but leads to a degree of over-treatment in those who are not deficient. Additionally, different approaches would be required for designing interventions at the individual-level compared to population-level. Much of the EED research to date has focused on population-level probiotic interventions targeting the gut microbiome of infants and children, particularly relating to treating diarrheal disease and growth stunting [1,52,53]. Studies have shown potential benefits of probiotics in infants and children with EED for these conditions [54–56], suggesting similar population-level approaches could be effective for pregnant women at risk of EED.

Overall, the pipeline of maternal gut microbiome candidates is small when compared to other maternal health conditions, such as pre-eclampsia and preterm labour/birth [25,26], with limited evidence available for all candidates [57]. However, analysis of this novel pipeline demonstrates there is a growing interest in this area of research. Despite the overall low number of candidates, there are a proportionately higher number of active candidates for maternal gut microbiome compared to other pregnancy-related conditions. Two-thirds of the candidates in the maternal gut microbiome pipeline commenced R&D within the previous five years, and almost 95% of R&D for all candidates began in the last ten years. The surge in R&D in recent years reflects an increased interest in the maternal gut microbiome that is likely to continue into the future.

Currently, the prevalence of EED in the general population and pregnant women is not known, in part due to the diagnostic barriers [12]. Despite limited diagnostics for EED, studies conducted in children with diarrheal disease and stunting show very high EED rates in populations with poor sanitation, high pathogen burden and chronic infections [1,52,53]. Although EED may not be regarded as a high-priority maternal condition like pre-eclampsia or postpartum haemorrhage (PPH), its impact on the gut microbiome likely influences the effectiveness of numerous interventions aimed at improving maternal outcomes. The detrimental effect of EED on the gut microbiome can reduce the absorption of critical nutrients, potentially undermining the efficacy of nutritional supplements such as iron, folic acid, and calcium, all of which are recommended for pregnant women [14,58]. This malabsorption can exacerbate general undernutrition and multi-nutrient deficiencies during pregnancy, in turn further affecting maternal outcomes. For example, anaemia, which is exacerbated by poor nutrient absorption, can increase the risk of PPH [59]. Therefore, if probiotics targeting the maternal gut microbiome prove effective in addressing EED, they could also prevent various pregnancy-related conditions and improve newborn and infant outcomes.

### Clinical and research implications

The complexity of the gut microbiome, influenced by factors such as nutrition and the environment [60], suggests that probiotics are unlikely to be a ‘silver bullet’ intervention that resolves all gut microbiome-related issues. A multifaceted public health approach, combining interventions such as probiotics (and pre- or post-biotics), alongside nutritional and environmental interventions will likely be more effective and sustainable in altering gut microbiome composition [61]. Additionally, as there is a vast diversity of probiotics with many different strains and combinations of strains [62], as well as different target mechanisms (e.g., inflammation vs pathogen suppression), the efficacy of probiotics is both strain-specific and disease-specific [63]. This indicates no single probiotic strain would effectively address all pregnancy-related conditions. Individualised analysis of the gut microbiome to develop personalised treatments is being explored outside of the maternal health space and may help to address this challenge [64]. Further evidence, including through basic research, is needed to identify the most promising strains or combinations for improving a range of maternal, foetal and newborn outcomes.

Importantly, gut microbiome composition differs across geographical regions and ethnicities [65–67]. A greater understanding of these differences is essential for developing region- or population-specific probiotic products. Notably, nearly all trials identified in the pipeline, as well as most preclinical studies, were conducted in high-income countries, highlighting a significant gap in understanding the impact of probiotics across diverse population groups. In addition, the gut microbiome undergoes changes during pregnancy [47,68,69]. Should an effective probiotic product be discovered, formative research would be needed in diverse contexts to understand local feasibility and acceptability, facilitating effective implementation.

Key research questions remain to facilitate the development of a probiotic product that meets the parameters of the TPP. Given the clinical efficacy of probiotics on maternal, foetal and infant outcomes is not yet known for all candidates in the pipeline, efficacy trials are required before these candidates can be considered for treating EED. To date, systematic reviews have found no benefits of probiotics during pregnancy [70–72], though available evidence is limited, much of which is low certainty. Additionally, safety data are critical. While the safety of probiotic use during pregnancy has generally been shown,[73] a Cochrane review found an increased risk of pre-eclampsia in overweight and obese pregnant women with probiotic use compared to placebo (RR 1.85, 95% CI 1.04 to 3.29; 4 studies, 955 women; high‐certainty evidence)[35]. In contrast, a 2024 systematic review found no evidence of increased pre-eclampsia risk with probiotic use during pregnancy [70]. Future trials should measure and minimise potential harms.

Despite the current lack of trial evidence on the effects of probiotics on pregnancy-related outcomes, and a dearth of trials in pregnant women with EED, there is considerable interest in this field [47]. There are efforts underway to develop a diagnostic test suitable for use during pregnancy, with a range of different technologies being explored [1,5,74,75]. Biomarkers are particularly promising - many potential biomarkers are being explored for use in EED diagnostics [74,76–79]. Ideally, an EED diagnostic test would be simple to use, non-invasive, available at point-of-care in antenatal settings and affordable. This would allow treatment interventions to be targeted to the individual woman, rather than requiring population-wide strategies. As an emerging and complex area, where much is still not fully understood, creating a rigorous and structured approach to research into EED in pregnant women will help to focus efforts on the highest potential areas [47,80]. This TPP, R&D pipeline and candidate matching approach can better guide all stakeholders involved and provide rigour and structure to this field of research.

### Strengths and limitations

This is the first TPP developed for interventions targeting the maternal gut microbiome. Systematic stakeholder mapping ensured diverse participants and views were obtained, in terms of geographical location, expertise and gender. This diversity, including strong representation from LMICs, ensures a balanced set of stakeholder perspectives and relevance of these outputs across various contexts. The R&D pipeline is the first database of its kind, mapping pre-clinical and clinical candidates for maternal microbiome interventions. Our TPP matching used a systematic, drug-agnostic approach, meaning high-potential candidates can be identified in an evidence-informed, unbiased fashion.

The current R&D pipeline is small, particularly in comparison to similar pipelines for other maternal health conditions [81]. Additionally, references to specific genus, such as *Lactobacillus spp.,* encompasses all species within that genus. As clinical effects of probiotics are species- and strain-specific, this generalisation limits the ability to extrapolate results across different species or strains within the genus. One challenge was finding enough participants with experience in maternal microbiome interventions. For example, no responses were received through the public consultation webpage. As an emerging field, there are relatively few individuals working in this disease area. To counteract this, we contacted many stakeholders through systematic identification methods. However, it is possible that additional responses may have led to differing agreement levels for some variables. Unlike some other published TPPs, we conducted a single round of interviews. However, given the feedback we received, and the degree of consensus amongst stakeholders, we do not consider that further rounds would significantly impact these findings. Considering the rapidly evolving nature of this field, it may be necessary to update the TPP and R&D pipeline in future to reflect ongoing innovation and developments.

## Conclusions

Development of this novel TPP showed agreement amongst diverse stakeholders as to most characteristics a probiotic intervention targeting the maternal gut microbiome should have. The TPP will assist in guiding key stakeholders working in maternal microbiome research to develop, trial and administer probiotic interventions that are accessible, affordable and effective for pregnant women globally. We identified one high- and two medium-potential probiotic candidates in the R&D pipeline. However, the lack of information available on candidates due to the emerging nature of clinical research into probiotics targeting the maternal microbiome suggests further evidence is needed. This combined method of TPP development, pipeline analysis, and candidate matching will inform and guide future research in probiotic interventions targeting the maternal gut microbiome.

## Supporting information

S1 Table Target Product Profile for Microbial Interventions (Probiotics) during pre-conception, pregnancy, and lactation to promote maternal health. (PDF)

S2 Table Strategies to identify stakeholders from each group. (DOCX)

S3 Table Distribution of stakeholders by WHO global region and gender. (DOCX)

S4 Table Data fields for pipeline. (DOCX)

S5 Table TPP candidates with matching weights and scoring definitions. (DOCX)

S6 Table Maternal microbiome interventions pipeline. (DOCX)
