# Supplementary material for: Maternal gut microbiome interventions to improve maternal and perinatal health outcomes: Target product profile expert consensus and pipeline analysis
Source: PLoS One. 2025 Jul 2;20(7):e0321543. doi: 10.1371/journal.pone.0321543 (PMC12221072; doi:10.1371/journal.pone.0321543)
Supplement: S1 Table — (PDF) [file pone.0321543.s001.pdf]

Target Product Profile for Microbial Interventions (Probiotics) during pre-conception, pregnancy, and lactation to promote maternal health

V2.2 September 2023

AIM
ACCELERATING INNOVATION FOR MOTHERS

Burnet

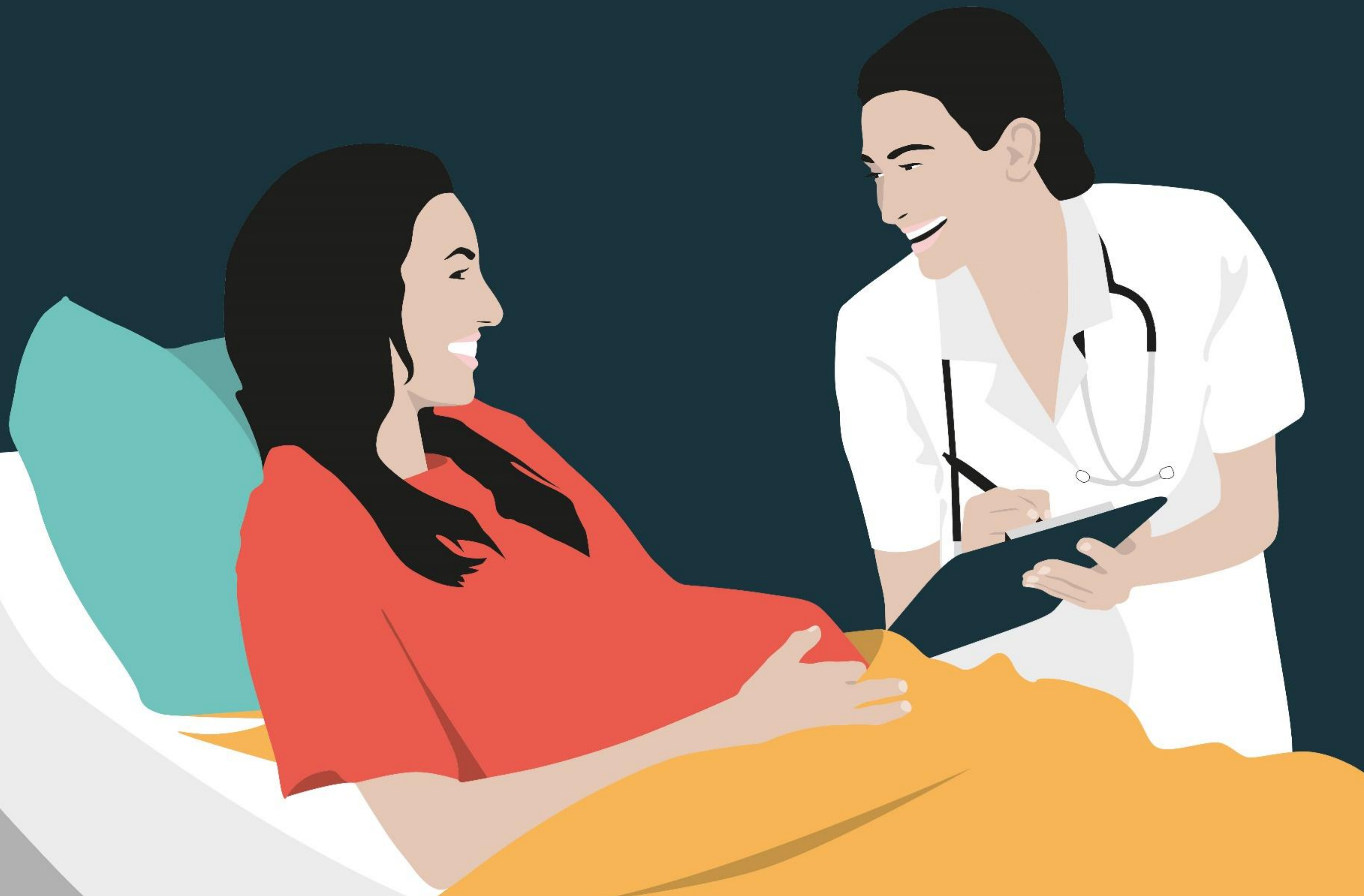

Glossary

Environmental Enteric Dysfunction (EED)	"An incompletely defined syndrome of inflammation, reduced absorptive capacity, and reduced barrier function in the small intestine."[1] EED is characterised by "enteric inflammation, villus blunting and decreased crypt-to-villus ratio."[2]
Gut dysbiosis	"Unhealthy imbalance in microbial composition"[3]
Gut microbiome	"The collection of bacteria, archaea and eukarya colonising the GI tract"[4]
Prebiotic	"A substrate that is selectively utilized by host microorganisms conferring a health benefit"[5]
Probiotic	"Live microorganisms which when administered in adequate amounts confer a health benefit on the host"[6]
Undernutrition	"Insufficient intake of energy and nutrients to meet an individual's needs to maintain good health"[7]

1. BACKGROUND

1.1 MATERNAL GUT MICROBIOME AND ENVIRONMENTAL ENTERIC DYSFUNCTION

The gut microbiome has been identified as an important, yet not entirely understood, factor influencing maternal, fetal and infant health outcomes.[8,9] The human gut microbiome (made up of predominantly bacteria, but also viruses, archaea and eukaryotic microbes[10]) influences many physiological functions including metabolic functions, immune system regulation, prevention of infection, and inflammatory responses.[11] An altered gut microbiome in a state of dysbiosis – defined as an "unhealthy imbalance in microbial composition"[3] – plays a role in multiple diseases and conditions, such as gastrointestinal disorders, colorectal cancer, diabetes, polycystic ovary syndrome, metabolic syndrome, mental health conditions and cardiovascular disease.[12,13] Gut microbiome dysbiosis can be influenced by multiple nutritional and environmental factors during pregnancy,[9,14,15] though limited evidence exists on typical pregnancy-related gut microbiome changes.[9] Available evidence presents conflicting findings - some studies suggest increased dysbiosis is common during pregnancy, possibly due to factors such as hormonal changes, weight gain and dietary changes,[16,17] while others show minimal to no gut microbiome alterations during pregnancy.[18,19] Additionally, composition of the gut microbiome appears to differ across different geographical locations and ethnicities.[20,21]

Environmental enteric dysfunction (EED) is characterized by gastrointestinal dysbiosis associated with subclinical inflammation in the small intestine, likely caused by subclinical enteropathogen infection – this causes poor macro/micronutrient absorption capacity, altered gut morphology and impaired barrier function.[22] There is limited evidence on the prevalence of EED in different groups, including pregnant women, in part due to the challenges of diagnosing EED.[23] However it is believed to be particularly common across populations in low- and middle-income countries.[24] For example, a growing body of evidence has demonstrated a high prevalence of EED in young children in LMICs,[25] that is associated with altered growth trajectories such as stunting.[26]

Environmental pathogen exposure seems to increase the risk of EED; the prevalence is hypothesized to be greater in regions where access to clean water and sanitation is limited.[2] Poor water, sanitation and hygiene results in frequent exposure to enteric pathogens, leading to chronic or recurrent infections.[27] Specific risk factors include lack of access to latrines, reliable clean water connections, storage of fecal matter near households, poor hand hygiene, contamination of groundwater, and proximity to livestock and animals.[27-30] Chronic or recurrent infections, including parasitic infections, can damage the gut barrier structure and function thereby contributing to EED. Infections may cause chronic inflammatory responses as well as structural changes that hinder intestinal nutrient absorption and lead to pathogen leakage.[1,31,32]

Maternal undernutrition, while not a direct cause of EED, can be indicative of high rates of EED. Maternal undernutrition is highly prevalent in many low- and middle-income countries (LMICs), particularly African and South Asian countries,[33] and contributes to an estimated 3.5 million deaths of mothers and children under five annually worldwide.[34] Gut microbiome-related conditions, such as EED and irritable bowel

syndrome, can impair the body's ability to absorb and efficiently process nutrients,[35,36] thereby contributing to undernutrition.

It is hypothesized that microbial dysbiosis is associated with adverse pregnancy outcomes, including preterm birth, gestational diabetes, hypertension, early-onset preeclampsia, low birthweight and stillbirth.[25,37,38] Preliminary evidence from studies involving fecal microbiota transplants from women diagnosed with preeclampsia into mice models have shown a manifestation of preeclampsia-related signs and symptoms.[39,40] Furthermore, a study employing fecal microbiota transplants on pregnant mice from women with gestational diabetes demonstrated increases in blood glucose levels.[41] Maternal, neonatal and infant outcomes can also be influenced by other microbiomes, particularly the vaginal and oral microbiomes.[8]

Though the relationship between the gut microbiome, EED and adverse pregnancy outcomes is complex, therapeutics that can prevent or treat maternal EED may have beneficial effects on maternal and newborn outcomes.

1.2 CURRENT PRODUCTS

There are currently limited effective treatment options for EED. Recent clinical research has focused on anti-inflammatory drugs, dietary supplements and antimicrobial interventions, mostly in malnourished children.[2] Several different types of microbial interventions exist, aimed at optimizing gut microbiome with varying mechanisms of action, modes of administration, and evidence of efficacy and acceptability. Some interventions take a targeted approach aiming to influence (either increase or decrease) specific microbial strains or taxa, whereas other interventions act more broadly, aiming to shape the composition of the entire 'community' of gut microbiota.[42]

Many of these interventions aim to improve maternal systemic or intestinal inflammation and gut permeability, or reduce pathogen burden and gut dysbiosis – these may be linked to poor maternal and infant outcomes. Some microbial therapeutics may potentially augment (or be adjunct to) other therapies, such as when administered following antibiotic therapy to reduce the risks of anti-microbial resistance and decreased gut microbiome diversity.[43,44] Microbiome-altering products may thus have the potential to enhance health outcomes for women and their babies.

This TPP focuses on microbial interventions – probiotic supplements and drugs. Probiotic supplements are defined as "live microorganisms which when administered in adequate amounts confer a health benefit on the host".[6] There are many types of probiotics, which can be administered as supplements. Commonly used bacterial genera in probiotics include *Lactobacillus, Bifidobacterium, Escherichia, Enterococcus, Bacillus* and *Streptococcus*.[45] Probiotics are also found in some foods, such as yoghurt and sauerkraut,[46] however probiotic-containing foods are beyond the scope of this TPP. Prebiotics are also not within the scope of this TPP, however could potentially supplement probiotics (either alone or when combined in synbiotics) to nourish beneficial gut bacteria.[47]

Alternatively, probiotics may be regulated as drugs, when registered for the treatment of a specific disease. These can contain either a single or multiple microbial strains, and their functionalities may arise from genetic engineering or through innate processes of the microbes.[48] A key distinction between these

products lies in their label claim: probiotic supplements aim to provide general health benefits, whereas probiotic drugs are intended to treat specific conditions or diseases.[49]

1.3 PURPOSE OF THIS TARGET PRODUCT PROFILE ON MICROBIAL INTERVENTIONS (PROBIOTICS)

Target Product Profiles (TPPs) are strategic documents that outline the minimum and optimal characteristics required for new health products, including medicines and devices. TPPs are an important resource to guide key stakeholders (such as funders, researchers, product developers, manufacturers and regulators) on the requirements of new medicines, diagnostics and devices to meet pre-specified clinical and public health needs.[50] They inform research and development strategies, help frame product dossiers, streamline communication with regulatory agencies and help funders set targets.[51]

Interventions that target maternal microbiome composition have the potential to correct gut dysbiosis, which has been associated with maternal undernutrition, and other complications of pregnancy.[15,52] The use of microbiome-altering interventions to correct gut dysbiosis in pregnancy and postpartum is an emerging area of research.[38,42] If interventions targeting the maternal gut microbiome and alleviating EED were shown to be safe and effective in improving maternal and infant health outcomes, it could help prevent the associated sequelae of maternal gut dysbiosis and EED.

There are currently no TPPs publicly available for microbiome-altering interventions in maternal health. Development of this TPP is intended to help drive innovation, research and implementation of effective interventions that can alter the maternal enteric microbiome, to improve outcomes of mothers and babies globally.

2. SUMMARY: INTERVENTION USE CASE AND TARGET USERS

A probiotic supplement or drug that targets the maternal gut microbiome in pre-conception, pregnant and lactating women, girls and trans and gender diverse people who are able to get pregnant. The product should impact at least one of the following: maternal gut and systemic inflammation, gut permeability, pathogen burden or microbiome composition or function that are linked to maternal (e.g. gestational diabetes, hypertension, obesity, preeclampsia, maternal infection) and/or infant (e.g. small for gestational age, preterm birth, low birth weight, sepsis, NEC, wasting, stunting) outcomes. Interventions should be affordable, and self-administered non-invasively.

The health worker cadre responsible for offering the intervention may depend on the specific product and country, but could include obstetricians, nurses, midwives, nutritionists/dietitians, general practitioners or pharmacists. Probiotic supplements can be taken orally by an individual woman, without the need for a trained healthcare professional to prepare and administer the product. Probiotic drugs are also taken orally, however they typically require a trained health professional to diagnose the condition to be treated and to prescribe the appropriate drug for that condition.

To ensure the correct target user group has widespread access to any effective microbial interventions, targeted distribution through established healthcare systems would be needed. The interventions should be incorporated into settings or facilities where routine antenatal care and nutrition programs are provided, particularly in settings where there is an increased risk of EED, such as where maternal undernutrition, high environmental pathogen exposure (due to poor water, sanitation and hygiene), and chronic infections are prevalent.

3. TARGET PRODUCT PROFILE

Variable	**Minimum**<br>*The minimal target should be considered as a potential go/no go decision point.*	**Optimistic**<br>*The optimistic target should reflect what is needed to achieve broader, deeper, quicker global health impact.*	**Annotations / Actual Product Performance**<br>*For all parameters, include here the rationale for why this feature is important and/or for the target value.*
Indication Investigated	<u>Supplement:</u><br>Probiotic supplement administered to promote gut health for women during preconception, pregnancy and lactation in settings with an increased risk of Environmental Enteric Dysfunction (EED).<br><br><u>Drug:</u><br>Probiotic therapeutic specifically treating women with suspected or confirmed EED.	Same as minimum	Probiotics supplements are intended to target general gut health in women with EED. However, there is currently no agreed upon diagnostic test for EED, which presents challenges to implementation.<br><br>Therefore, probiotic supplements are indicated at the regional or population-level for women in settings with an increased risk of EED. Risk factors for EED include regions or settings with high rates of undernutrition, environmental pathogen exposure (due to poor water, sanitation and hygiene) and chronic infections. These risk factors for EED are informed by risk factors used in trials for EED treatments in children.[25,27,28,30] Other risk factors may yet exist.<br><br>Probiotic drugs, as a therapeutic product, are indicated for individual women with EED, though this necessitates further advancements in EED diagnostics.

Variable	**Minimum**<br>*The minimal target should be considered as a potential go/no go decision point.*	**Optimistic**<br>*The optimistic target should reflect what is needed to achieve broader, deeper, quicker global health impact.*	**Annotations / Actual Product Performance**<br>*For all parameters, include here the rationale for why this feature is important and/or for the target value.*
Target Population	Pregnant women, lactating women, or women/adolescents of reproductive age preparing for conception, living in settings with a high risk of EED.	Pregnant women, lactating women, or women/adolescents of reproductive age preparing for conception, living in settings with a high risk of EED, or who have suspected or diagnosed EED.	EED may affect women prior to, during or after pregnancy. There is not yet consensus on the diagnosis of EED. It can potentially be diagnosed through invasive tests such as endoscopy and small intestinal biopsy (which are contraindicated during pregnancy),[1] or laboratory diagnostic tests such as stool testing or organic acid (urine) testing.[53,54] Biomarker tests (e.g. dual-sugar absorption tests) are an emerging alternative diagnostic option,[2] however further innovation is needed to strengthen available diagnostic options. Despite having less resource-intensive requirements in comparison to invasive tests,[55] biomarker tests may still have limited availability in LMICs.[56]<br><br>Due to these challenges, the target population can be defined by settings with an increased risk of EED including those with high burdens of undernutrition, environmental pathogen exposure (due to poor water, sanitation and hygiene) and chronic infections.[25,27,28,30]
Special populations	Must be safe and effective for use in women and adolescents of all body compositions, including those who are underweight and overweight/obese.	Same as minimum	Products must be safe and effective for use in all women (pregnant and non-pregnant) who have any degree of severity of undernutrition.[57] Products must also be safe for overweight or obese women. Women taking these products

Variable	Minimum<br>*The minimal target should be considered as a potential go/no go decision point.*	Optimistic<br>*The optimistic target should reflect what is needed to achieve broader, deeper, quicker global health impact.*	Annotations / Actual Product Performance<br>*For all parameters, include here the rationale for why this feature is important and/or for the target value.*
	Safe for women with common conditions of pregnancy such as gestational diabetes and hypertension.		may not have a sufficient macro and/or micronutrient intake.
Population unlikely to be treated	Not intended for women with a medical contraindication to the intervention, such as immunosuppression.	Same as minimum	Women who have a specific contraindication to probiotics would not be suitable to receive the intervention.
Target Countries	Low- and middle-income countries with an increased population-level risk of EED.	All low-, middle- and high-income countries.	While data are limited, EED is believed to be highly prevalent in low- and middle-income countries (LMICs), due to a high burden of factors associated with an increased risk of EED, including undernutrition, environmental pathogen exposure and chronic infections.[1,33] EED may also be prevalent in high-income countries, particularly in populations experiencing systemic disadvantage.[58]
Clinical Efficacy Outcomes	Reduced gut or systemic inflammatory markers<br><br>OR<br><br>Reduced enteropathogen abundance<br><br>OR<br><br>Improved pregnancy and/or birth outcomes	Reduced gut or systemic inflammatory markers<br><br>OR<br><br>Reduced enteropathogen abundance<br><br>OR	Measuring reductions in inflammatory markers generally relies on proxy biomarker tests, however there is not yet consensus on which tests should be used. Biomarker tests commonly used in studies of EED in children include the lactulose:mannitol (L:M) dual sugar test and various fecal and blood biomarkers of gut and systemic inflammation.[23,59-63] Additionally, some tests may not be readily available, appropriate or of adequate quality in many countries, particularly lower-resource countries.[64,65]

Variable	Minimum<br>*The minimal target should be considered as a potential go/no go decision point.*	Optimistic<br>*The optimistic target should reflect what is needed to achieve broader, deeper, quicker global health impact.*	Annotations / Actual Product Performance<br>*For all parameters, include here the rationale for why this feature is important and/or for the target value.*
		Improved pregnancy and/or birth outcomes<br><br>OR<br><br>Improved breastmilk composition<br><br>OR<br><br>Improved infant growth and/or other health outcomes	Minimum efficacy outcomes reflect the reduction in inflammatory markers, enteropathogen burden, and outcomes such as low birth weight and preterm birth which may be indicative of improved nutritional status in undernourished women.[66] Optimistic efficacy outcomes also include enhanced composition of breastmilk beneficial to infants, as well as infant health and development outcomes.
Safety	Clinical safety (adverse or serious adverse effects for mother and baby) comparable to current therapies.<br><br>Not contraindicated in pregnant and lactating women.<br><br>Absence of fetal and embryonic toxicity or teratogenicity.<br><br>No risk of overdose if more than the recommended dose is consumed.<br><br>Manufacture of product with consistent quality that meets global minimum standards and national registration requirements, in particular label accuracy.	Fewer adverse effects than current therapies.<br><br>Not contraindicated in pregnant and lactating women.<br><br>Absence of fetal and embryonic toxicity or teratogenicity.<br><br>No risk of overdose if more than the recommended dose is consumed.<br><br>Manufacture of product with consistent quality that meets global minimum standards and national registration requirements, in particular label accuracy.	Probiotic supplements are generally considered safe for use during preconception, pregnancy and lactation.[67-69] A forthcoming systematic review has found no evidence of benefit or harm relating to maternal, fetal or newborn outcomes, though this evidence is largely low- or very low-certainty.[70]<br><br>Evidence on the use of probiotic drugs to treat EED during pregnancy and lactation is currently limited. Establishing the safety profile of probiotic drugs for these populations must consider the influence of diverse microbial strains in drugs, as well as potential interactions with the host microbiome.[71]

Variable	Minimum<br>*The minimal target should be considered as a potential go/no go decision point.*	Optimistic<br>*The optimistic target should reflect what is needed to achieve broader, deeper, quicker global health impact.*	Annotations / Actual Product Performance<br>*For all parameters, include here the rationale for why this feature is important and/or for the target value.*
		Evidence shows no drug-related long-term or serious adverse effects for mothers or babies.	Current comparable therapies for probiotic supplements could include micronutrient supplementation such as iron folic acid (IFA) or multiple micronutrient supplementation (MMS). Currently, there are no drugs indicated for treatment of EED in pregnant women that would present an appropriate comparison.<br><br>The International Probiotics Association has developed Probiotic Manufacturing Guidelines[72] to supplement Good Manufacturing Practice (GMP) guidance.[73]
Need for clinical monitoring	Standard continued monitoring of maternal, fetal and infant health and wellbeing, as per usual antenatal and newborn care practices.<br><br>Minimal additional monitoring required for known product side-effects (such as gastrointestinal upset and skin reactions), consistent with the time period in which such events may arise as a direct consequence of product administration.	Same as minimum	Regular assessment of maternal and fetal wellbeing and growth is recommended during pregnancy.[74] Global guidelines exist on antenatal care,[74] however standard monitoring practices may differ across different countries and contexts.[75]<br><br>Common side effects of probiotics include gastrointestinal side effects and skin complications.[76,77]
Is companion diagnostic needed for use?	Supplement:<br>No individual diagnostic is needed for an intervention targeted to contexts with increased risk of EED.	Supplement:<br>No individual diagnostic is needed for an intervention targeted to contexts with increased risk of EED.	Population-level interventions that do not require EED diagnostics, but instead focus on settings with a high risk of EED, are more feasible to implement in LMICs.

Variable	Minimum<br>*The minimal target should be considered as a potential go/no go decision point.*	Optimistic<br>*The optimistic target should reflect what is needed to achieve broader, deeper, quicker global health impact.*	Annotations / Actual Product Performance<br>*For all parameters, include here the rationale for why this feature is important and/or for the target value.*
	<u>Drug:</u><br>Additional diagnostic needed to confirm EED to ensure appropriate target population/individual is being treated.	<u>Drug:</u><br>Additional diagnostic needed to confirm EED to ensure appropriate target population/individual is being treated.<br><br>Optimally, the development of a diagnostic test for EED that is affordable, accessible, non-invasive and simple to use would be beneficial to implementation in LMICs.	In contrast, targeting the intervention to a specific population with EED may increase effectiveness and efficiency of treatments. However, there is currently no globally agreed diagnostic test or criteria for EED.[78] Additionally, EED is often asymptomatic.[1] As such, diagnoses of EED is often complex and inaccurate, typically requiring invasive, stool, blood or urine tests.[1,53,54]
Label Claim	<u>Supplement:</u><br>Supports and promotes good gut health.<br><br><u>Drug:</u><br>Supports in the treatment of EED.	<u>Supplement:</u><br>Supports and promotes good gut health.<br><br><u>Drug:</u><br>Supports in the treatment of EED.<br><br>*Plus*<br>Improvements in maternal and/or neonatal outcomes (e.g. gestational diabetes, birthweight, preterm birth).	Label claims reflect the indication of probiotic supplements for general gut health benefits, and drugs to target EED. Optimally, this also includes a reduction in adverse maternal and neonatal outcomes.
Product format, administration, frequency, and dose	Oral administration, preferably capsules or tablets.<br><br>Daily doses.	Oral administration, including capsules, tablets, pre-portioned powder sachets or gummies that do not require reconstitution with	These interventions are most commonly administered orally when targeting the gut microbiome.[79,80] Oral administration would likely

Variable	Minimum *The minimal target should be considered as a potential go/no go decision point.*	Optimistic *The optimistic target should reflect what is needed to achieve broader, deeper, quicker global health impact.*	Annotations / Actual Product Performance *For all parameters, include here the rationale for why this feature is important and/or for the target value.*
	Acceptable and tolerable dose.<br><br>Can be administered during pre-conception, all trimesters of pregnancy and lactation.	water or other additional ingredients.<br><br>Daily doses.<br><br>Acceptable and tolerable dose, with consideration of appropriate taste, texture, and colour.<br><br>Can be administered during pre-conception, all trimesters of pregnancy and lactation.	be acceptable and feasible in limited-resource settings.<br><br>Daily doses are likely preferable in most contexts, with benefits to compliance if taken regularly, particularly alongside other daily supplements (e.g. iron folic acid). Evidence is needed to determine optimal frequency, duration and dosing schedule for optimising efficacy.<br><br>Probiotics may be more effective when administered in conjunction with other interventions such as improved nutritional quality and quantity and increased access to safe water and sanitation.[81]
Drug interactions	No known significant drug-drug interactions with common antenatal treatments (medicines or supplements), food products (such as nutrient supplementation, fortified or supplementary foods), or drugs used for common comorbidities of gut dysbiosis.	Same as minimum.	The treatment must have minimal to no adverse interactions with drugs commonly used in pre-conception, pregnant or lactating women with gut dysbiosis, EED or common conditions of pregnancy. Examples include supplements such as iron, folic acid, calcium, vitamin B, multiple micronutrient supplementation (MMS), and foods naturally containing probiotics. Any potential drug interactions with antibiotics must be considered, with administration advice for specific products provided accordingly.

Variable	Minimum<br>*The minimal target should be considered as a potential go/no go decision point.*	Optimistic<br>*The optimistic target should reflect what is needed to achieve broader, deeper, quicker global health impact.*	Annotations / Actual Product Performance<br>*For all parameters, include here the rationale for why this feature is important and/or for the target value.*
			Gut dysbiosis is believed to be associated with an increased risk of several conditions including inflammatory bowel disease, obesity, autoimmune diseases, type I and II diabetes, chronic kidney disease, polycystic ovary syndrome, metabolic syndrome and mental health conditions.[82-85] Women with these conditions may be taking other medications as part of their management.
Stability / Shelf Life	Stable at 30°C.<br><br>Easy to transport and store in a range of climatic conditions, including humidity and heat, without affecting potency over time.<br><br>24-36 month shelf life as per product storage instructions.<br><br>No requirement for cold chain.	Stable at 30°C, or preferably higher temperatures.<br><br>Easy to transport and store in a range of climatic conditions, including humidity and heat, without affecting potency over time.<br><br>3 to 5-year shelf life in climatic zone IVb (simulated with 30°C and 75% relative humidity).<br><br>No requirement for cold chain.	Products must be suitable for use in various temperature and climate conditions, particularly given the climate of many LMICs.<br><br>Some probiotic supplements and drugs require cold chain, however increasingly products are being developed that are suitable for transport and storage at room temperature.[86,87]
Product Registration and Regulation	Approval from national regulatory agency in target country.	Same as minimum.	Probiotics supplements would not require registration or regulation through a drug

Variable	Minimum<br>*The minimal target should be considered as a potential go/no go decision point.*	Optimistic<br>*The optimistic target should reflect what is needed to achieve broader, deeper, quicker global health impact.*	Annotations / Actual Product Performance<br>*For all parameters, include here the rationale for why this feature is important and/or for the target value.*
	Supplement:<br>Registration through supplement pathway.<br><br>Drug:<br>Registration through drug pathway.<br><br>Approval by at least one internationally recognized regulatory authority (e.g. USFDA, European Medicines Agency, Swissmed).		pathway, avoiding the complexities associated with this pathway.<br><br>Probiotic drugs specifically targeting EED would need to be registered through a drug pathway given their therapeutic nature.[49]<br><br>All interventions must be approved by the national regulatory authority in the relevant country.
Product presentation	Easy to open and consume.<br><br>Packaging should be opaque and aim to protect and preserve the quality of the product and prevent degradation or damage during transport and storage.<br><br>Information on strain/s, dosage and instructions for use included on label.	Compact. Easy to open and consume.<br><br>Packaging should be opaque and aim to protect and preserve the quality of the product and prevent degradation or damage during transport and storage.<br><br>Information on strain/s, dosage and instructions for use included on label.<br><br>Packaging adapted for local context, including name, colours and images used.	Packaging that is easy to open and simple for women to take will aid in the implementation of these products.<br><br>For probiotic drugs, packaging and design must comply with regulatory guidance from a stringent regulatory authority or WHO standards for packaging for pharmaceutical products.[88]

Variable	Minimum<br>*The minimal target should be considered as a potential go/no go decision point.*	Optimistic<br>*The optimistic target should reflect what is needed to achieve broader, deeper, quicker global health impact.*	Annotations / Actual Product Performance<br>*For all parameters, include here the rationale for why this feature is important and/or for the target value.*
		Environmental impact of the packaging should be minimized.<br><br>Child-resistant packaging to prevent unintended use by children.	
Primary Target Delivery Channel	Appropriately trained health workers in a range of settings including:<br>- Formal antenatal and postnatal services where pregnant women are receiving care.<br>- Routine health facility services for women of reproductive age for pre-conception, including community-based outreach services and women's health clinics.<br>- Nutrition programs targeting undernutrition.<br><br><u>Supplement:</u><br>Additionally, non-health facility delivery channels such as community pharmacies or supermarkets for 'over-the-counter' access without a prescription.	Same as minimum	The intervention should be easily delivered in a range of health facilities, including local primary facilities.<br><br>Probiotic drugs would require suitably trained health professionals to diagnose EED and prescribe a suitable drug.<br><br>In contrast, probiotic supplements delivered as a population-level intervention would not require a health professional to diagnose a specific condition or provide a prescription, so could also be delivered over-the-counter through other channels such as pharmacies, supermarkets, etc.
Target Procurement Price	Products are affordable in the public sector in LMICs. Where possible, products should be available free of charge to women.	Products are affordable in the public sector in LMICs. Where possible, products should be available free of charge to women.	Affordability is an essential consideration, particularly given the burden of undernutrition and other EED risk factors in LMICs and limited resource settings.[33] Affordability is essential to

Variable	Minimum<br>*The minimal target should be considered as a potential go/no go decision point.*	Optimistic<br>*The optimistic target should reflect what is needed to achieve broader, deeper, quicker global health impact.*	Annotations / Actual Product Performance<br>*For all parameters, include here the rationale for why this feature is important and/or for the target value.*
		Unit cost of products is similar to other treatments for women with EED or commonly used maternal supplements such as multiple micronutrient supplementation (MMS).<br><br>Bulk purchase discounts available for organizations, nutrition programs or governments.	individual consumers and for larger scale procurements by governments or organizations. Local or regional manufacturing has potential to bring down costs and ensure products are locally acceptable.
Expected financing sources	Procurement in LMICs financed by national governments, international agencies (including UN organizations), and/or international donors, or private sector.	Procurement financed by national governments or private sector.	Procurement of medicines for use in pregnancy, lactation and for women of reproductive age in LMICs varies between countries. It may include governments as well as support from international organizations, agencies or funders.<br><br>Procurement of effective treatments for EED would ideally be prioritized by national governments.
Volume estimates	Volumes compatible with target population (preconception, pregnancy and lactation) in settings with a high burden of EED risk	Same as minimum	There are currently no reliable global estimates of EED in pregnant or lactating women, or women of reproductive age.

Variable	Minimum<br>*The minimal target should be considered as a potential go/no go decision point.*	Optimistic<br>*The optimistic target should reflect what is needed to achieve broader, deeper, quicker global health impact.*	Annotations / Actual Product Performance<br>*For all parameters, include here the rationale for why this feature is important and/or for the target value.*
	factors, including undernutrition, environmental pathogen exposure due to poor water, sanitation and hygiene, and chronic infections.		The estimated prevalence of low BMI in women across Africa and Asia is greater than 10%.[33] In Africa, malnutrition among pregnant women is estimated at 23.5%,[89] with rates in some areas significantly higher, including between 38% to 44.9% in several regions of Ethiopia.[90,91]<br><br>An estimated 3.6 billion people globally do not have access to safely managed sanitation facilities.[92] Approximately 2.2 billion people globally are without safely managed drinking-water services.[93]<br><br>As maternal EED is an emerging research area, there are currently no reliable global estimates on the coverage of probiotic supplements or drugs in the target populations.

4. REFERENCES

1. Crane RJ, Jones KJ, Berkley JA. Environmental enteric dysfunction: An overview. *Food and Nutrition Bulletin* 2015; **36**(1): 76-87.
2. Tickell K, Atlas H, Walson J. Environmental enteric dysfunction: a review of potential mechanisms, consequences and management strategies. *BMC Medicine* 2019; **17**.
3. Edwards S, Cunningham S, Dunlop A, Corwin E. The Maternal Gut Microbiome During Pregnancy. *MCN Am J Matern Child Nurs* 2017; **42**(6): 310-7.
4. Thursby E, Juge N. Introduction to the human gut microbiota. *Biochem J* 2017; **474**(11): 1823-36.
5. Gibson G, Hutkins R, Sanders M, Prescott S, et al. Expert consensus document: The International Scientific Association for Probiotics and Prebiotics (ISAPP) consensus statement on the definition and scope of prebiotics. *Nat Rev Gastroenterol Hepatol* 2017; **14**(8): 491-502.
6. Hill C, Guarner F, Reid G, Gibson G, et al. Expert consensus document. The International Scientific Association for Probiotics and Prebiotics consensus statement on the scope and appropriate use of the term probiotic. *Nat Rev Gastroenterol Hepatol* 2014; **11**(8): 506-14.
7. Maleta K. Undernutrition. *Malawi Med J* 2006; **18**(4): 189-205.
8. Dunlop A, Mulle J, Ferranti E, et al. Maternal Microbiome and Pregnancy Outcomes That Impact Infant Health. *Advances in Neonatal Care* 2015; **15**(6): 377-85.
9. Sinha T, Brushett S, Prins J, Zhernakova A. The maternal gut microbiome during pregnancy and its role in maternal and infant health. *Current Opinion in Microbiology* 2023; **74**.
10. Shreiner A, Kao J, Young V. The gut microbiome in health and in disease. *Curr Opin Gastroenterol* 2015; **31**(1): 69-75.
11. Bander Z, Nitert M, Mousa A, Naderpoor N. The Gut Microbiota and Inflammation: An Overview. *Int J Environ Res Public Health* 2020; **17**(20): 7618.
12. Bull M, Plummer N. Part 1: The Human Gut Microbiome in Health and Disease. *Integrative Medicine* 2014; **13**(6): 17-22.
13. Hills R, Pontefract B, Mishcon H, Black C, Sutton S, Theberge C. Gut Microbiome: Profound Implications for Diet and Disease. *Nutrients* 2019; **11**(7): 1613.
14. Maher S, O'Brien E, Moore R, et al. The association between the maternal diet and the maternal and infant gut microbiome: a systematic review. *British Journal of Nutrition* 2020; **129**(9).
15. Strobel K, Juul S, Hendrixson D. Maternal Nutritional Status and the Microbiome across the Pregnancy and the Post-Partum Period. *Microorganisms* 2023; **11**(6): 1569.
16. Koren O, Goodrich J, Cullender T, et al. Host Remodeling of the Gut Microbiome and Metabolic Changes during Pregnancy. *Cell Reports* 2012; **150**: 470-80.
17. Nuriel-Ohayon M, Neuman H, Ziv O, et al. Progesterone Increases Bifidobacterium Relative Abundance during Late Pregnancy. *Cell Reports* 2019; **27**: 730-6.
18. Yang H, Guo R, Li S, et al. Systematic analysis of gut microbiota in pregnant women and its correlations with individual heterogeneity. *npj Biofilms and Microbiomes* 2020; **6**.
19. DiGiulio D, Callahan B, McMurdie P, Relman D. Temporal and spatial variation of the human microbiota during pregnancy. *Microbiology* 2015; **112**(35): 11060-5.
20. Porras A, Shi Q, Zhou H, et al. Geographic differences in gut microbiota composition impact susceptibility to enteric infection. *Cell Reports* 2021; **36**(4).

21. Dwiyanto J, Hussain M, Reidpath D, et al. Ethnicity influences the gut microbiota of individuals sharing a geographical location: a cross-sectional study from a middle-income country. *Scientific Reports* 2021; **11**.
22. Moya-Alvarez V, Sansonetti PJ. Understanding the pathways leading to gut dysbiosis and enteric environmental dysfunction in infants: the influence of maternal dysbiosis and other microbiota determinants during early life. *FEMS Microbiol Rev* 2022; **46**(3).
23. Mahfuz M, Das S, Mazumder R, et al. Bangladesh Environmental Enteric Dysfunction (BEED) study: protocol for a community-based intervention study to validate non-invasive biomarkers of environmental enteric dysfunction. *BMJ Open* 2017; **7**(8): e017768.
24. Cowardin C, Syed S, Iqbal N, et al. Environmental enteric dysfunction: gut and microbiota adaptation in pregnancy and infancy. *Nature* 2023; **20**: 223-37.
25. Lauer JM, Duggan CP, Ausman LM, et al. Biomarkers of maternal environmental enteric dysfunction are associated with shorter gestation and reduced length in newborn infants in Uganda. *Am J Clin Nutr* 2018; **108**(4): 889-96.
26. Gizaw Z, Yalew A, Bitew B, et al. Stunting among children aged 24–59 months and associations with sanitation, enteric infections, and environmental enteric dysfunction in rural northwest Ethiopia. *Scientific Reports* 2022; **12**.
27. George C, Oldja L, Biswas S, et al. Fecal Markers of Environmental Enteropathy Are Associated with Animal Exposure and Caregiver Hygiene in Bangladesh. *Am J Trop Med Hyg* 2015; **93**(2): 269-75.
28. Wang A, Shulman R, Crocker A, et al. A Combined Intervention of Zinc, Multiple Micronutrients, and Albendazole Does Not Ameliorate Environmental Enteric Dysfunction or Stunting in Rural Malawian Children in a Double-Blind Randomized Controlled Trial. *The Journal of Nutrition* 2017; **147**(1): 97-103.
29. Mbuya M, Humphrey J. Preventing environmental enteric dysfunction through improved water, sanitation and hygiene: an opportunity for stunting reduction in developing countries. *Maternal & Child Nutrition* 2015; **12**(1): 106-20.
30. Exum N, Lee G, Olórtegui M, et al. A Longitudinal Study of Household Water, Sanitation, and Hygiene Characteristics and Environmental Enteropathy Markers in Children Less than 24 Months in Iquitos, Peru. *Am J Trop Med Hyg* 2018; **98**(4): 995-1004.
31. Abou-Seri H, Abdalgaber M, Zahran F. Enteric parasitic infections: From environmental enteric dysfunction to gut microbiota and childhood malnutrition. *Parasitologists United Journal* 2022; **15**(3): 216-23.
32. Gabain I, AS. R, Webster J. Parasites and childhood stunting – a mechanistic interplay with nutrition, anaemia, gut health, microbiota, and epigenetics. *Trends in Parasitology* 2023; **39**(3).
33. Black RE, Victoria CG, Walker SP, Bhutta ZA, et al. Maternal and child undernutrition and overweight in low-income and middle-income countries. *Lancet* 2013; **382**(9890): 427-51.
34. The Lancet. The Lancet's Series on Maternal and Child Undernutrition Executive Summary, 2008.
35. Montoro-Huguet M, Belloc B, Dominguez-Cajal M. Small and Large Intestine (I): Malabsorption of Nutrients. *Nutrients* 2021; **13**(4): 1254.
36. Jandhyala S, Talukdar R, Subramanyam C, et al. Role of the normal gut microbiota. *World J Gastroenterol* 2015; **21**(29): 8787-803.
37. Kirby M, Lauer J, Muhini A, et al. Biomarkers of environmental enteric dysfunction and adverse birth outcomes: An observational study among pregnant women living with HIV in Tanzania. *eBioMedicine* 2022; **84**.

38. Di Simone N, Santamaria Ortiz A, Specchia M, et al. Recent Insights on the Maternal Microbiota: Impact on Pregnancy Outcomes. *Front Immunol* 2020; **11**: 528202.
39. Chen X, Li P, Liu M, et al. Gut dysbiosis induces the development of preeclampsia through bacterial translocation. *Gut* 2020; **69**: 513-22.
40. Jin J, Gao L, Zou X, et al. Gut Dysbiosis Promotes Preeclampsia by Regulating Macrophages and Trophoblasts. *Circulation Research* 2022; **131**: 492-506.
41. Qin S, Wang Y, Wang S, et al. Gut microbiota in women with gestational diabetes mellitus has potential impact on metabolism in pregnant mice and their offspring. *Frontiers in Microbiology* 2022; **13**.
42. Hitch T, Hall L, Walsh S, et al. Microbiome-based interventions to modulate gut ecology and the immune system. *Mucosal Immunol* 2022; **15**: 1095-113.
43. Doan T, Hinterwirth A, Arzika A, et al. Mass Azithromycin Distribution and Community Microbiome: A Cluster-Randomized Trial. *Open Forum Infectious Diseases* 2018; **5**(8).
44. McDonnell L, Gilkes A, Ashworth M, Rowland V, et al. Association between antibiotics and gut microbiome dysbiosis in children: systematic review and meta-analysis. *Gut Microbes* 2021; **13**(1): 1-18.
45. Gupta V, Garg R. Probiotics. *Indian J Med Microbiol* 2009; **27**(3): 202-9.
46. Syngai G, Gopi R, Bharali R, et al. Probiotics - the versatile functional food ingredients. *J Food Sci Technol* 2016; **53**(2): 921-33.
47. Markowiak P, Śliżewska K. Effects of Probiotics, Prebiotics, and Synbiotics on Human Health. *Nutrients* 2017; **9**(9).
48. Heavey M, Durmusoglu D, Crook N, Anselmo A. Discovery and delivery strategies for engineered live biotherapeutic products. *Trends in Biotechnology* 2022; **40**(3): 354-69.
49. Cordaillat-Simmons M, Rouanet A, Pot B. Live biotherapeutic products: the importance of a defined regulatory framework. *Experimental & Molecular Medicine* 2020; **52**: 1397-406.
50. Food and Drug Administration. Guidance for Industry and Review Staff Target Product Profile — A Strategic Development Process Tool (Draft Guidance). 2007.
51. Tyndall A, Du W, Breder CD. Regulatory watch: The target product profile as a tool for regulatory communication: advantageous but underused. *Nat Rev Drug Discov* 2017; **16**(3): 156.
52. Gorczyca K, Obuchowska A, Kimber-Trojnar Z, et al. Changes in the Gut Microbiome and Pathologies in Pregnancy. *Int J Environ Res Public Health* 2022; **19**(16): 9961.
53. Chen L, Reynolds C, David R, Brewer A. Development of an Index Score for Intestinal Inflammation-Associated Dysbiosis Using Real-World Stool Test Results. *Digestive Diseases and Sciences* 2020; **65**(4): 1111-24.
54. Lord R, Bralley J. Clinical applications of urinary organic acids. Part 2. Dysbiosis markers. *Altern Med Rev* 2008; **13**(4): 292-306.
55. Ordiz M, Davitt C, Stephenson K, et al. EB 2017 Article: Interpretation of the lactulose:mannitol test in rural Malawian children at risk for perturbations in intestinal permeability. *Experimental Biology and Medicine* 2018; **243**(8): 677-83.
56. PATH. Market Failures and Opportunities for Increasing Access to Diagnostics in Low- and Middle-Income Countries. Seattle: PATH, 2022.
57. Whelan K, Myers C. Safety of probiotics in patients receiving nutritional support: a systematic review of case reports, randomized controlled trials, and nonrandomized trials. *The American Journal of Clinical Nutrition* 2010; **91**(3): 687-703.
58. Kukuruzovic R, Brewster D. Small bowel intestinal permeability in Australian aboriginal children. *J Pediatr Gastroenterol Nutr* 2002; **35**(2): 206-12.

59. Denno D, VanBuskirk K, Nelson Z, et a;. Use of the Lactulose to Mannitol Ratio to Evaluate Childhood Environmental Enteric Dysfunction: A Systematic Review. *Clinical Infectious Diseases* 2014; **59**(4): 213-9.
60. Kosek M, The MAL-ED Network Investigators. Causal Pathways from Enteropathogens to Environmental Enteropathy: Findings from the MAL-ED Birth Cohort Study. *eBioMedicine* 2017; **18**: 109-17.
61. Kosek M, Haque R, Lima A, et al. Fecal markers of intestinal inflammation and permeability associated with the subsequent acquisition of linear growth deficits in infants. *Am J Trop Med Hyg* 2013; **88**(2): 390-6.
62. Iqbal M, Sadiq K, Syed S, et al. Promising Biomarkers of Environmental Enteric Dysfunction: A Prospective Cohort study in Pakistani Children. *Scientific Reports* 2018; **8**.
63. Gizaw Z, Yalew A, Bitew B, et al. Fecal biomarkers of environmental enteric dysfunction and associated factors among children aged 24–59 months in east Dembiya district, northwest Ethiopia. *BMC Gastroenterology* 2022; **22**.
64. Wilson M, Fleming K, Kuti M, et al. Access to pathology and laboratory medicine services: a crucial gap. *Lancet* 2018; **391**(10133): 1927-38.
65. Nkengasong J, Yao K, Onyebujoh P. Laboratory medicine in low-income and middle-income countries: progress and challenges. *Lancet* 2018; **391**(10133): 1873-5.
66. da Silva Lopes K, Ota E, Shakya P, et al. Effects of nutrition interventions during pregnancy on low birth weight: an overview of systematic reviews. *BMJ Global Health* 2017; **2**.
67. Sheyholislami H, Connor K. Are Probiotics and Prebiotics Safe for Use during Pregnancy and Lactation? A Systematic Review and Meta-Analysis. *Nutrients* 2021; **13**(7): 2382.
68. Obuchowska A, Gorczyca K, Standyło A, et al. Effects of Probiotic Supplementation during Pregnancy on the Future Maternal Risk of Metabolic Syndrome. *Int J Mol Sci* 2022; **23**(15): 8253.
69. Tsakiridis I, Kasapidou E, Daglis T, et al. Nutrition in Pregnancy: A Comparative Review of Major Guidelines. *Obstet Gynecol Surv* 2020; **75**(11): 692-702.
70. Nguyen R, McDougall A, Vogel J, et al. The effect of probiotics administration during pregnancy on preeclampsia and associated maternal, fetal and newborn outcomes - a systematic review and meta-analysis. 2023. https://www.crd.york.ac.uk/prospero/display_record.php?ID=CRD42023421613 (accessed 26 September 2023).
71. Rouanet A, Bolca S, Bru A, Claes I, et al. Live Biotherapeutic Products, A Road Map for Safety Assessment. *Frontiers in Medicine* 2020; **7**(237).
72. International Probiotics Association. Probiotic Manufacturing Guidelines. 2019. https://internationalprobiotics.org/wp-content/uploads/IPA-Probiotic-Manufacturing-Guidelines-2019.pdf (accessed 7 September 2023).
73. World Health Organization. Guidelines: Norms and Standards for Pharmaceuticals. 2023. https://www.who.int/teams/health-product-and-policy-standards/standards-and-specifications/norms-and-standards-for-pharmaceuticals/guidelines (accessed 7 September 2023).
74. World Health Organization. WHO recommendations on antenatal care for a positive pregnancy experience. WHO: Geneva; Switzerland, 2016.
75. Kruizenga H, Beijer S, Huisman-de Waal G, et al. Guideline on Malnutrition: Recognising, Diagnosing and Treating Malnutrition in Adults: Dutch Malnutrition Steering Group, 2017.
76. Didari T, Solki S, Mozaffari S, et al. A systematic review of the safety of probiotics. *Expert Opin Drug Saf* 2014; **13**(2): 227-39.

77. Sotoudegan F, Daniali M, Hassani S, et al. Reappraisal of probiotics' safety in human. *Food Chem Toxicol* 2019; **129**: 22-9.
78. Denno D, Tarr P, Nataro J. Environmental Enteric Dysfunction: A Case Definition for Intervention Trials. *Am J Trop Med Hyg* 2017; **97**(6): 1643-6.
79. Han S, Lu Y, Xie J, et al. Probiotic Gastrointestinal Transit and Colonization After Oral Administration: A Long Journey. *Front Cell Infect Microbiol* 2021; **11**.
80. Balfour H. Developing and delivering live biotherapeutic products. *European Pharmaceutical Review* 2021; (5).
81. Arnold B, Null C, Luby S, et al. Cluster-randomised controlled trials of individual and combined water, sanitation, hygiene and nutritional interventions in rural Bangladesh and Kenya: the WASH Benefits study design and rationale *BMJ Open* 2013; **3**.
82. Vijay A, Valdes A. Role of the gut microbiome in chronic diseases: a narrative review. *European Journal of Clinical Nutrition* 2022; **76**(489-501).
83. DeGruttola A, Low D, Mizoguchi A, Mizoguchi E. Current Understanding of Dysbiosis in Disease in Human and Animal Models. *Inflammatory Bowel Diseases* 2016; **22**(5): 1137-50.
84. Sun Y, Gao S, Ye C, Zhao W. Gut microbiota dysbiosis in polycystic ovary syndrome: Mechanisms of progression and clinical applications. *Front Cell Infect Microbiol* 2023; **13**.
85. Wang P, Deng X, Zhang C, Yuan H. Gut microbiota and metabolic syndrome. *Chin Med J* 2020; **133**(7): 808-16.
86. Fenster K, Freeburg B, Hollard C, et al. The Production and Delivery of Probiotics: A Review of a Practical Approach. *Microorganisms* 2019; **7**(3): 83.
87. Charbonneau M, Isabella V, Li N, Kurtz C. Developing a new class of engineered live bacterial therapeutics to treat human diseases. *Nature Communications* 2020; **11**: 1738.
88. WHO Health Product and Policy Standards. Technical Report Series No. 902 - 36th report of the WHO Expert Committee on Specifications for Pharmaceutical Preparations. Annex 9 Guidelines on packaging for pharmaceutical products. Geneva, Switzerland: World Health Organization, 2002.
89. Desyibelew H, Dadi A. Burden and determinants of malnutrition among pregnant women in Africa: A systematic review and meta-analysis. *PLoS One* 2019; **14**(9).
90. Arero G. Undernutrition and associated factors among pregnant women in East Borena Zone, Liban District, Oromia regional state, Ethiopia. *Frontiers in Nutrition* 2022; **9**.
91. Chea N, Tegene Y, Astatkie A, Spigt M. Prevalence of undernutrition among pregnant women and its differences across relevant subgroups in rural Ethiopia: a community-based cross-sectional study. *J Health Popul Nutr* 2023; **42**(17).
92. The World Bank. Sanitation. 2022. https://www.worldbank.org/en/topic/sanitation (accessed 15 September 2023).
93. World Health Organization. Drinking-water. 2023. https://www.who.int/news-room/fact-sheets/detail/drinking-water (accessed 15 September 2023).
