# Supplementary material for: Maternal gut microbiome interventions to improve maternal and perinatal health outcomes: Target product profile expert consensus and pipeline analysis
Source: PLoS One. 2025 Jul 2;20(7):e0321543. doi: 10.1371/journal.pone.0321543 (PMC12221072; doi:10.1371/journal.pone.0321543)
Supplement: S2 Table — (DOCX) [file pone.0321543.s002.docx]

**Supplemental Table 2: Strategies to identify stakeholders from each group**

| ***Stakeholder groups*** | ***# Key Stakeholder***  ***Interviews*** | ***Methods of stakeholder identification*** |
| --- | --- | --- |
| Obstetricians with special interest/expertise in maternal microbiome or nutrition | 3 | - Via listservs of special societies/professional groups (FIGO, FLASOG, AOFOG and AFOG). - WHO professional networks with OBGYN societies internationally |
| Midwives and nurses | 3 | - Via listservs of international nursing and midwifery societies (ICM, INF and others). |
| Academics / researchers working on maternal microbiome | 3 | - Systematic identification of top 100 researchers through SCOPUS, with direct email contact |
| Dietitian/Nutritionists | 3 | - Via listervs of relevant special societies/international groups (e.g. World Public Health Nutrition Association (WPHNA), International Affiliate of the Academy of Nutrition and Dietetics (IAAND), International Confederation of Dietetic Associations (ICDA), Scaling Up Nutrition (SUN)) |
| Antenatal care program managers | 3 | - Via WHO QED Network (11 countries) and WHO Antenatal Care Implementation Research sites (4 countries) - Through WHO Country Offices, for further distribution to National Ministry of Health’s ANC program manager networks |
| Biotech and nutraceutical manufacturers | 3 | - Master list of biotech and nutraceutical companies developed through literature review, and to a list of biotech and nutraceutical manufacturers held by WHO Procurement |
| Consumer representatives | 3 | - To Partnership on Maternal, Newborn and Child Health network organizations - Online searching to further develop list of consumer organizations interested in maternal microbiome/undernutrition |
| Guideline panel members | 3 | - Literature review to identify and contact all guideline panel members from WHO, NICE, USA and other relevant guidelines relating to maternal microbiome and maternal nutrition from the last 10 years |
| Procurement experts | 3 | - Via WHO and UNICEF contact lists of procurement officers, and national ministry of health procurement agencies |
| International health agency or organization staff | 3 | - To WHO, UNICEF and UNFPA staff working on maternal nutrition at Headquarters, Regional and Country Office levels - Web searches to identify other international non-governmental organizations, with direct emails sent |
