# Supplementary material for: Maternal gut microbiome interventions to improve maternal and perinatal health outcomes: Target product profile expert consensus and pipeline analysis
Source: PLoS One. 2025 Jul 2;20(7):e0321543. doi: 10.1371/journal.pone.0321543 (PMC12221072; doi:10.1371/journal.pone.0321543)
Supplement: S3 Table — (DOCX) [file pone.0321543.s003.docx]

**Supplemental Table 3: Distribution of stakeholders by WHO global region and gender**

| **Stakeholder group** | **Interview participants  n=23 (%)** | **Survey participants  n=32 (%)** |
| --- | --- | --- |
| *Obstetricians* | 3 (13.0%) | 4 (11.1%) |
| *Midwives/Nurses* | 2 (8.7%) | 5 (13.9%) |
| *Academics* | 5 (21.7%) | 18 (50.0%) |
| *Dietician/Nutritionist* | 3 (13.0%) | - |
| *Antenatal care/nutrition  programme managers* | 1 (4.3%) | 1 (2.8%) |
| *Biotech and nutraceutical manufacturers* | 2 (8.7%) | 1(2.8%) |
| *Guideline panel members* | 1 (4.3%) | 1 (2.8%) |
| *Procurement expert* | 2 (8.7%) | - |
| *International health organisation staff* | 2 (8.7%) | 4 (11.1%) |
| *Consumer representative* | 2 (8.7%) | - |
| *Other* | - | 2 (5.6%) |
| **Gender** |  |  |
| *Woman* | 16 (70%) | 20 (63%) |
| *Man* | 7 (30%) | 11 (34%) |
| *Prefer to self-describe* | - | 1 (3%) |
| *Prefer not to say* | - | - |
| **Geographic location** |  |  |
| *African region* | 5 (21.7%) | 3 (9.4%) |
| *Region of the Americas* | 5 (21.7%) | 5 (15.6%) |
| *Eastern Mediterranean region* | 2 (8.7%) | 1 (3.1%) |
| *European region* | 3 (13.0%) | 6 (18.8%) |
| *South-East Asian region* | 5 (21.7%) | 2 (6.3%) |
| *Western Pacific region* | 3 (13.0%) | 15 (46.9%) |
