# Supplementary material for: Maternal gut microbiome interventions to improve maternal and perinatal health outcomes: Target product profile expert consensus and pipeline analysis
Source: PLoS One. 2025 Jul 2;20(7):e0321543. doi: 10.1371/journal.pone.0321543 (PMC12221072; doi:10.1371/journal.pone.0321543)
Supplement: S4 Table — (DOCX) [file pone.0321543.s004.docx]

**Supplemental Table 4: Data fields for pipeline**

| **Name** |
| --- |
| **Alternative names** |
| **Pregnancy-specific condition** |
| **Product type** |
| **Subproduct type** |
| **Indication type (prevention and/or treatment)**  **Indication** |
| **Archetype (new chemical entity or repurposed)** |
| **Target** |
| **Route of administration** |
| **Mechanism of action** |
| **Medical subject headings (MeSH)** |
| **Key features** |
| **Clinical use status (investigational or marketed)** |
| **Current R&D stage** |
| **Highest R&D stage for any condition** |
| **Development status (active or inactive)** |
| **Inactive development reason (if applicable)** |
| **Regional specificity of product** |
| **Location of R&D** |
| **Developers** |
| **Known funders** |
| **Preclinical results status** |
| **Preclinical results type** |
| **Preclinical and clinical results sources** |
| **Clinical trials:**   - - **CT title**   - **CT number**   - **CT last updated**   - **CT phase**   - **CT source**   - **CT status**   - **CT terminated type**   - **CT terminated reason**   - **CT start type**   - **CT start date**   - **CT end type**   - **CT end date**   - **CT description**   - **CT locations**   - **CT results status**   - **CT results type**   - **CT results source**   - **CT sponsors**   - **CT collaborators** |
