# Supplementary material for: Maternal gut microbiome interventions to improve maternal and perinatal health outcomes: Target product profile expert consensus and pipeline analysis
Source: PLoS One. 2025 Jul 2;20(7):e0321543. doi: 10.1371/journal.pone.0321543 (PMC12221072; doi:10.1371/journal.pone.0321543)
Supplement: S5 Table — (DOCX) [file pone.0321543.s005.docx]

**Supplemental Table 5: TPP candidates with matching weights and scoring definitions**

| **Candidate name** | **Target Population** | **Target Country** | **Efficacy** | **Companion diagnostics** | **Clinical monitoring** | **Safety** | **Format & Administration** | **Stability** | **Final rank** |
| --- | --- | --- | --- | --- | --- | --- | --- | --- | --- |
| Probiotic combination - four unspecified strains | Not yet known | HIC only | Not yet known | Not yet known | Not yet known | Not yet known | Met optimal | Unsure | LOW |
| Vivomixx *(De Simone formulation – 8 strains)* | Met optimal | HIC and LMIC | Not yet known | Met minimum | Met optimal | Met optimal | Met optimal | Yes | HIGH |
| Probiotic combination - unspecified strains | Not yet known | LMIC only | Not yet known | Not yet known | Not yet known | Not yet known | Partially met minimum | Unsure | LOW |
| Probiotics and LC-PUFA - combined, unspecified strains | Not yet known | LMIC only | Not yet known | Not yet known | Not yet known | Not yet known | Not yet known | Unsure | LOW |
| *Lactobacillu*s and *Bifidobacterium* - combined | Not yet known | HIC only | Not yet known | Not yet known | Not yet known | Not yet known | Met optimal | No | LOW |
| *Lactobacillus spp.* | Partially met minimum | HIC and LMIC | Not yet known | Not yet known | Not yet known | Not yet known | Met optimal | No | MEDIUM |
| *Bifidobacterium spp.* | Not yet known | HIC and LMIC | Not yet known | Not yet known | Not yet known | Met optimal | Met optimal | No | MEDIUM |

***spp. - species. Dark green = met optimum; light green = met minimum; yellow = partially met minimum; red = did not meet minimum; grey = not yet known.***

| **Candidate name** | **Target Population** | **Target Country** | **Efficacy** | **Companion diagnostics** | **Clinical monitoring** | **Safety** | **Format & Administration** | **Stability** | **Final rank** |
| --- | --- | --- | --- | --- | --- | --- | --- | --- | --- |
| Probiotic combination - four unspecified strains | 0 | 1 | 0 | 0 | 0 | 0 | 3 | 0 | 4 |
| Vivomixx *(De Simone formulation – 8 strains)* | 3 | 2 | 0 | 2 | 3 | 3 | 3 | -2 | 14 |
| Probiotic combination - unspecified strains | 0 | 1 | 0 | 0 | 0 | 0 | 1 | 0 | 2 |
| Probiotics and LC-PUFA - combined, unspecified strains | 0 | 1 | 0 | 0 | 0 | 0 | 0 | 0 | 1 |
| *Lactobacillus* and *Bifidobacterium* - combined | 0 | 1 | 0 | 0 | 0 | 0 | 3 | 2 | 6 |
| *Lactobacillus spp.* | 1 | 2 | 0 | 0 | 0 | 0 | 3 | 2 | 8 |
| *Bifidobacterium spp.* | 0 | 2 | 0 | 0 | 0 | 3 | 3 | 2 | 10 |

| **Variable** | **Variable description** | **Answer options** | **Coding value** |
| --- | --- | --- | --- |
| *Target population* | In the available trials, was the included population pregnant women in high-risk environmental enteric dysfunction (EED) settings? | Not yet known | 0 |
|  |  | Not met minimum | -2 |
|  |  | Partially met minimum | 1 |
|  |  | Met minimum | 2 |
|  |  | Met optimum | 3 |
| *Target country* | Has the drug been trialled in high income countries only or at least one low- and middle-income country? | Not stated | 0 |
|  |  | HIC only | 1 |
|  |  | LMIC only | 1 |
|  |  | HIC and LMIC | 2 |
| *Clinical Efficacy Outcomes* | In the available trials, has the drug demonstrated clinically significant effect on the efficacy outcome/s? | Not yet known | 0 |
|  |  | Not met minimum | AUTOMATIC LOW |
|  |  | Partially met minimum | 1 |
|  |  | Met minimum | 3 |
|  |  | Met optimum | 5 |
| *Safety* | In the available trials, has the drug demonstrated any safety concerns? | Not yet known | 0 |
|  |  | Not met minimum | AUTOMATIC LOW |
|  |  | Partially met minimum | 1 |
|  |  | Met minimum | 2 |
|  |  | Met optimum | 3 |
| *Clinical Monitoring* | In the available trials, has the drug required the use of routine OR additional clinical monitoring? | Not yet known | 0 |
|  |  | Not met minimum | -2 |
|  |  | Partially met minimum | 1 |
|  |  | Met minimum | 2 |
|  |  | Met optimum | 3 |
| *Companion Diagnostic* | In the available trials, has the drug required the routine use of a companion diagnostic test? | Not yet known | 0 |
|  |  | Not met minimum | -2 |
|  |  | Partially met minimum | 1 |
|  |  | Met minimum | 2 |
|  |  | Met optimum | 3 |
| *Product Format, Administration, Frequency and Dose* | What is the product format, administration, frequency and dose of the candidate? | Not yet known | 0 |
|  |  | Not met minimum | -2 |
|  |  | Partially met minimum | 1 |
|  |  | Met minimum | 2 |
|  |  | Met optimum | 3 |
| *Stability/Cold Chain* | Is cold chain required for this product? | Yes | -2 |
|  |  | No | 2 |
|  |  | Unsure | 0 |
