# Supplementary material for: Maternal gut microbiome interventions to improve maternal and perinatal health outcomes: Target product profile expert consensus and pipeline analysis
Source: PLoS One. 2025 Jul 2;20(7):e0321543. doi: 10.1371/journal.pone.0321543 (PMC12221072; doi:10.1371/journal.pone.0321543)
Supplement: S6 Table — (DOCX) [file pone.0321543.s006.docx]

**Supplemental Table 6: Maternal microbiome interventions pipeline**

| **PCR ID** | **Name** | **Product type** | **Sub-product** | **Archetype** | **Current R&D stage** | **Development status** |
| --- | --- | --- | --- | --- | --- | --- |
| 4581 | Akkermansia muciniphila | Microbial interventions | Probiotics | Repurposed | Discovery and preclinical | Active |
| 5881 | Probiotic combination - four unspecified strains | Microbial interventions | Probiotics | Repurposed | Phase I | Active |
| 5010 | Vivomixx | Microbial interventions | Probiotics | Repurposed | Phase II | Active |
| 5882 | Probiotic combination - unspecified strains | Microbial interventions | Probiotics | Repurposed | Phase II | Active |
| 5038 | Probiotics and LC-PUFA - combined, unspecified strains | Microbial interventions | Probiotics | Repurposed | Phase II | Active |
| 5036 | *Lactobacillus* and *Bifidobacterium* - combined | Microbial interventions | Probiotics | Repurposed | Phase II | Inactive |
| 4578 | *Lactobacillus spp.* | Microbial interventions | Probiotics | Repurposed | Phase II | Active |
| 5035 | *Bifidobacterium spp.* | Microbial interventions | Probiotics | Repurposed | Phase II | Active |
| 4575 | Biochemically diverse plant polysaccharides - unspecified | Bioactive compounds | Glycans | NCE | Discovery and preclinical | Active |
| 5884 | Plant-derived oligosaccharides (prebiotic phytochemical combination) | Bioactive compounds | Glycans | NCE | Discovery and preclinical | Active |
| 5008 | Galactooligosaccharide | Bioactive compounds | Glycans | Repurposed | Discovery and preclinical | Active |
| 6182 | Inulin | Bioactive compounds | Glycans | Repurposed | Discovery and preclinical | Active |
| 5003 | Prebiotic banana extract + Iron: Native Yogurt Banana (NaYOBA) | Bioactive compounds | Glycans | NCE | Phase II | Active |
| 5880 | Prebiotic-containing dairy - unspecified | Bioactive compounds | Glycans | Repurposed | Phase II | Active |
| 5006 | Oligosaccharide-sialic acid | Bioactive compounds | Glycans | Repurposed | Phase II | Inactive |
| 5007 | Fructooligosaccharide | Bioactive compounds | Glycans | Repurposed | Phase II | Active |
| 5001 | GOS/lcFOS - combined | Bioactive compounds | Glycans | Repurposed | Phase II | Inactive |
| 6183 | Microbiota-directed complementary food-2 (MDCF-2) | Bioactive compounds | Glycans | Repurposed | Phase II | Active |
| 4577 | Acetate | Bioactive compounds | Microbial metabolites | NCE | Discovery and preclinical | Inactive |
| 4579 | Butyrate | Bioactive compounds | Microbial metabolites | Repurposed | Discovery and preclinical | Active |
| 4580 | Propionate | Bioactive compounds | Microbial metabolites | NCE | Discovery and preclinical | Active |
| 5033 | Puerariae Lobatae Radix | Bioactive compounds | Polyphenols | Repurposed | Discovery and preclinical | Active |
| 5002 | Garlic oil | Bioactive compounds | Polyphenols | Repurposed | Discovery and preclinical | Active |
| 6181 | 919 Syrup | Bioactive compounds | Polyphenols | Repurposed | Discovery and preclinical | Active |
| 6184 | Omega-3 Fatty Acids | Bioactive compounds | Fatty acids | Repurposed | Phase II | Active |
| 5005 | Melatonin - MEM | Drugs |  | Repurposed | Discovery and preclinical | Active |
| 4574 | Sulfamethoxazole and trimethoprim - combined | Drugs |  | Repurposed | Phase II | Active |
| 5032 | Sulfadoxine and pyrimethamine - combined | Drugs |  | Repurposed | Phase II | Active |
| 5872 | Faecal microbiota transplant | Microbial interventions | FMT | Repurposed | Phase I | Active |
| 5889 | Fermented soy & dairy - unspecified | Microbial interventions | Fermented foods | Repurposed | Not applicable | Inactive |
| 4576 | Bifidobacterium fermented milk | Microbial interventions | Fermented foods | Repurposed | Phase II | Active |
| 5887 | Soymilk-Burkina (fermented milk and millet beverage) | Microbial interventions | Fermented foods | Repurposed | Phase II | Active |
| 5037 | Moringa and Lactobacillus rhamnosus GR-1 probiotic yogurt | Microbial interventions | Fermented foods | NCE | Phase II | Inactive |
| 5876 | Fermented millet porridge | Microbial interventions | Fermented foods | Repurposed | Phase II | Active |
| 5000 | Dadiah (fermented milk) | Microbial interventions | Fermented foods | Repurposed | Phase II | Active |
| 5034 | Torani (fermented rice water) | Microbial interventions | Fermented foods | Repurposed | Phase II | Active |
| 5885 | Mageu (fermented grain porridge) | Microbial interventions | Fermented foods | Repurposed | Phase II | Active |
| 5886 | Achars (pickles) | Microbial interventions | Fermented foods | Repurposed | Phase II | Active |
